# Neighborhood environments and transition to cognitive states: Sydney Memory and Ageing Study

**DOI:** 10.1002/alz.70569

**Published:** 2025-08-05

**Authors:** Ester Cerin, Annabel P. Matison, Miguel A. Molina, Ralf‐Dieter Schroers, Wei Li, Luke D. Knibbs, Vibeke Sorensen Catts, Yu‐Tzu Wu, Maria V. Soloveva, Kaarin J. Anstey, Suzanne Mavoa, Govinda Poudel, Bin Jalaludin, Nicole A. Kochan, Henry Brodaty, Perminder S. Sachdev

**Affiliations:** ^1^ Mary MacKillop Institute for Health Research Australian Catholic University Melbourne Victoria Australia; ^2^ School of Public Health The University of Hong Kong Hong Kong China; ^3^ Centre for Healthy Brain Ageing, School of Clinical Medicine University of New South Wales Sydney New South Wales Australia; ^4^ School of Public Health The University of Sydney Sydney New South Wales Australia; ^5^ Population Health Sciences Institute, Faculty of Medical Sciences, Campus for Ageing and Vitality Newcastle University Newcastle upon Tyne UK; ^6^ School of Psychology University of New South Wales Kensington New South Wales Australia; ^7^ Neuroscience Research Australia (NeuRA) Randwick New South Wales Australia; ^8^ UNSW Ageing Futures Institute University of New South Wales Kensington New South Wales Australia; ^9^ Population Health, Murdoch Children's Research Institute Royal Children's Hospital Parkville Victoria Australia; ^10^ Melbourne School of Population & Global Health University of Melbourne Carlton Victoria Australia; ^11^ School of Population Health University of New South Wales Kensington New South Wales Australia; ^12^ Neuropsychiatric Institute, The Euroa Centre Prince of Wales Hospital Randwick New South Wales Australia

**Keywords:** air pollution, blue space, built environment, dementia, green space, mild cognitive impairment

## Abstract

**INTRODUCTION:**

Features of the neighborhood environment and ambient air pollution have been associated with onset and progression of neurocognitive disorders, but data from longitudinal population‐based studies are limited.

**METHODS:**

One thousand thirty‐six participants (78.3 ± 4.8 years) of the Sydney Memory and Ageing Study were followed for up to 13.7 years with biennial cognitive assessments. Neighborhood environmental features were assessed around the participants’ homes. Associations between environmental features and transitions to cognitive states were estimated.

**RESULTS:**

Population density, street connectivity, access to commercial services, public transport, water bodies, and tree canopy were associated with a lower likelihood of worsening cognitive state. The opposite was observed for annual average concentrations of PM_2.5_. Access to parkland, blue spaces, and public transport were associated with a higher likelihood of reversal from mild cognitive impairment to normal cognition.

**DISCUSSION:**

Healthy neighborhood environments may delay cognitive decline and the onset of dementia in older individuals.

**Highlights:**

- This is the first published study on neighborhood built and natural environmental correlates of transition to dementia.
- This study was conducted in socially advantaged areas with relatively low ambient air pollution.
- Walkable neighborhoods are associated with a lower likelihood of worsening cognitive state.
- Neighborhood tree canopy is consistently predictive of better cognitive outcomes.
- Access to public transport, and blue and green spaces is associated with higher probability of improved cognitive state.

## 1 BACKGROUND

With > 57 million people living with dementia and the number forecast to triple by 2050,^1^ dementia is a global health issue. Mild cognitive impairment (MCI) that is not severe enough to meet a diagnosis of dementia is also a major contributor to disability and societal costs, the latter being estimated to be US$2,000 to $2500 per month per person.^2^ Because there is no cure for dementia and MCI, prevention remains the best strategy to tackle their impacts on individuals and society.

The Lancet Commission identified 14 modifiable risk factors estimated to be responsible for up to 45% of dementia cases globally.^3^ These include 13 person‐level demographic, disease, and lifestyle risk factors and a recently added environmental factor—namely, air pollution. The disproportionate representation of person‐level risk factors in the report may partially be the result of decades of exclusive research focus on “the individual” at the expense of the environment in which the individual lives. While a plethora of studies have identified characteristics of the neighborhood environment as important determinants of diseases (e.g., diabetes)^4^, ^5^ and lifestyle risk factors (e.g., physical inactivity)^6^, ^7^ associated with dementia,^3^ research on environmental determinants of dementia, and cognitive health in general, has been lagging.^8^, ^9^ This is unfortunate because the creation of neighborhoods that support healthy lifestyles is a key sustainable, long‐term, population‐wide strategy for the prevention of chronic non‐communicable diseases,^10^, ^11^ including dementia.^12^, ^13^, ^14^ Moreover, the importance of the neighborhood environment for cognitive health is even more pronounced in late life when people often spend most of their time in their neighborhood due to retirement and ill health^15^ and are at higher risk of dementia.

Associations between various physical features of the neighborhood environment and incidence of dementia and MCI have been reported.^8^, ^9^, ^16^, ^17^ In line with theoretical models,^9^, ^12^, ^18^ several studies suggest that better access to green space^9^, ^19^ and local services, such as retail and community centers,^9^, ^19^ may lower the risk of dementia/MCI, while exposure to air pollutants, especially particulate matter < 2.5 microns in diameter (PM_2.5_), increases the risk.^3^, ^20^ However, the evidence is still scant, sometimes mixed, and limited in scope and methodological rigor.^9^, ^20^ Specifically, most studies in this area adopted an overly simplistic theoretical framework typically focusing only on one of the three main dimensions of the neighborhood physical environment (built environment, natural environment, and environmental hazards, such as noise and air pollution) and ignoring the fact that some environmental features are causally related.^9^, ^12^ This is a serious oversight because, for example, the omission of key environmental confounders, such as population density, from models of ambient air pollution and incident dementia would likely attenuate the effect estimates of air pollution because population density is a source of air pollution as well as opportunities for an active lifestyle.^12^, ^21^ On the other hand, population density may emerge as a protective factor for dementia only after conditioning on ambient air pollution (one of the mediators on the pathway between population density and incident dementia).^19^

To our knowledge, just one cohort study with a 12‐year follow‐up period considered all three dimensions of the neighborhood environment and their causal interrelationships in association with incident dementia/MCI and reversal from MCI to normal cognition.^19^ The study focused on incident MCI and was conducted on a relatively young (60–64 years at baseline) and healthy population living in a relatively low‐density environment at baseline. The present study adds to the literature by expanding the investigation to incident dementia using data from a cohort of Australians aged 70+ years at baseline, living in a denser urban environment, and followed for up to 13.7 years. The objective of the study was to estimate the overall as well as independent effects (i.e., adjusted for potential environmental mediators) of all three dimensions of the neighborhood environment (built environment, natural environment, and air pollution) on transitions to MCI, dementia, and reversals from MCI to normal cognition.

## 2 METHODS

### 2.1 Study design and procedure

This study used data from the Sydney Memory and Ageing Study (Sydney MAS),^22^ which commenced in 2005 with participant recruitment from two electoral areas in Sydney's eastern suburbs. Participants were randomly selected from the electoral roll (registration is compulsory for Australian citizens). Inclusion criteria were English speaking, community‐dwelling adults aged 70 to 90 years, without dementia. Exclusion criteria included a previous diagnosis of dementia, psychotic symptoms, schizophrenia, bipolar disorder, multiple sclerosis, motor neuron disease, developmental disability, progressive malignancy, or any condition that may have prevented assessment completion. Participants undertook follow‐up assessments every ≈ 2 years for up to 13.7 years (total number of waves = 7; median follow‐up time: 9.7 years). Each wave included a neuropsychological assessment and self‐report of current residential address.

**RESEARCH IN CONTEXT**

1. **Systematic review**: Studies have typically examined only a few neighborhood features as correlates of older adults’ cognitive impairment, and those that considered all key features (built and natural environments, and pollution) were cross‐sectional and/or conducted on young‐old adults. We examined associations of all key neighborhood features with incident mild cognitive impairment (MCI) and dementia, and reversal from MCI to normal cognition, in a cohort of Australians aged ≥ 70 years.
2. **Interpretation**: Residents of neighborhoods with higher density, connectivity, tree cover access to commercial services, public transport, and blue spaces, and lower air pollution were less likely to experience clinically significant cognitive decline. Residents of neighborhoods with better access to public transport, parkland, and blue spaces were more likely to revert from MCI to normal cognition.
3. **Future directions**: Future research is warranted in diverse geographical and socio‐economic contexts to establish the generalizability of the findings and identify the mechanisms underpinning the observed associations.

Sydney MAS was approved by the ethics committees at University of New South Wales Sydney and the South Eastern Sydney Illawarra Area Health Service. The research was carried out in compliance with the principles outlined in the Declaration of Helsinki. All participants gave written informed consent.

### 2.2 Participants

**FIGURE 1:**
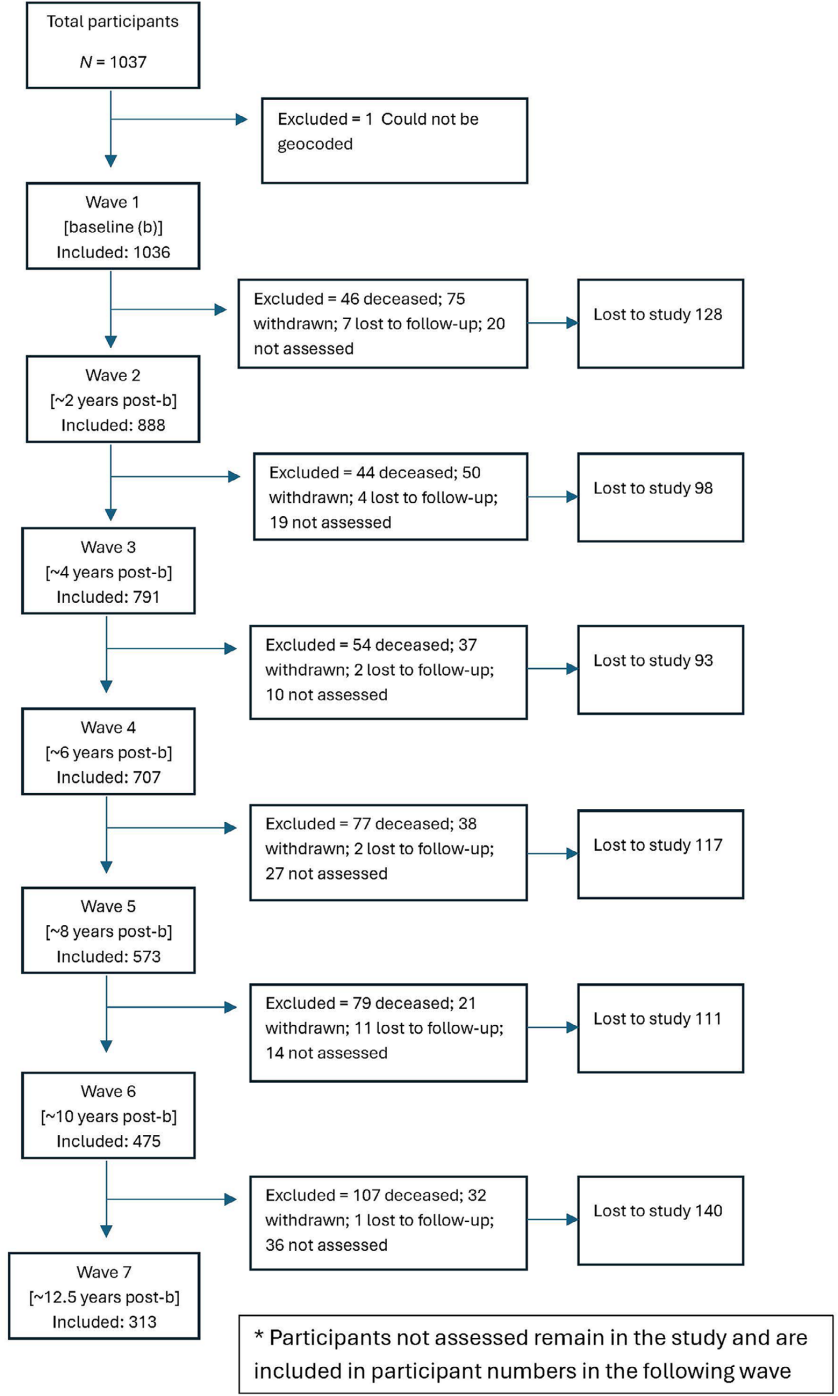
Flowchart of participants in the Sydney Memory and Ageing Study, assessed biennially, with the first assessment in Wave 1 occurring in September 2005 and the final assessment in Wave 7 occurring in November 2020.

Community‐dwelling individuals (*N* = 1037) aged 70 to 90 years, free from dementia, were recruited and successfully assessed at baseline (Wave 1). One of these participants had a residential address that could not be geocoded. Hence, the analytical sample for the present study included 1036 participants. Over the follow‐up period, 407 participants died, 253 withdrew from the study, 27 were lost to follow‐up, and 36 were not assessed at Wave 7, resulting in 313 participants included at Wave 7. Details of participant numbers and reasons for dropout from Wave 2 to Wave 7 are provided in Figure 1. Figure S1 in supporting information shows the geographical distribution of the participants at baseline (Wave 1) at the level of Statistical Areas 1 (SA1), small geographical areas used for Census purposes.

### 2.3 Measures

#### 2.3.1 Cognitive states (outcomes)

For Waves 1 to 4 participants’ cognitive function was assessed using a comprehensive neuropsychological battery, covering attention/processing speed (Digit Symbol Coding,^23^ Trail‐Making Test Part A^24^), executive function (Trail‐Making Test Part B,^24^ Controlled Oral Word Association Test^24^), language (Boston Naming Test,^25^ Semantic Fluency [Animals]^24^), visuospatial ability (Block Design Test^26^), and visual memory (Benton Visual Retention Test recognition^27^) and verbal memory (Logical Memory Story A delayed recall,^28^ Rey Auditory Verbal Learning Test^24^). Participants scoring 1.5 standard deviations below normative data on memory or non‐memory measures or showing reduced scores and a decline in daily or instrumental activities^29^, ^30^ were brought to a consensus meeting. Consensus diagnosis of MCI or dementia was made by at least three experienced clinicians from a panel of neuropsychiatrists, psychogeriatricians, and neuropsychologists, using international consensus criteria (MCI;^31^ dementia Diagnostic and Statistical Manual of Mental Disorders 4th Edition [DSM‐IV] criteria^32^) and information from clinical, neuropsychological, laboratory, and imaging data (when available). Participants who performed in the expected range on the neuropsychological tests according to normative data (adjusted for age and/or education) were deemed to have normal cognition. Participants were categorized as “unclassifiable” when it was considered that there was too much missing neuropsychological data (or issues with test interpretability—e.g., in non‐native English speakers) or missing activities of daily living data. Unclassifiable participants with a previous dementia diagnosis were assigned a dementia diagnosis.

**FIGURE 2:**
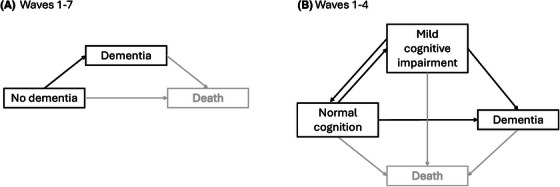
Transitions states used in multi‐state Markov models. Death was modeled as a state but was not the focus of this study.

For Waves 5 to 7, a similar process was followed using an abbreviated neuropsychological battery, with only the Modified Telephone Interview for Cognitive Status (TICS‐M^33^) used at Wave 5, and the Mini Mental State Examination (MMSE)^34^ and Addenbrooke's Cognitive Examination III (ACE‐III)^35^ used in Wave 6. Consensus diagnosis of either no dementia or dementia according to DSM‐IV criteria^32^ was determined for participants meeting criteria for review by the expert panel. Due to the limited cognitive assessment data collected, MCI was not assessed after Wave 4. Hence, we considered two sets of transition cognitive states: (1) no dementia, dementia (all waves) and (2) normal cognition, MCI, dementia (Waves 1–4; Figure 2).

#### 2.3.2 Socio‐demographic and health attributes (confounders or covariates)

Socio‐demographic attributes included as confounders or covariates in the analyses were: sex, age, English‐speaking background status (yes vs. no), years of education, living arrangements (living in the community with partner or others; living in the community alone; other living arrangements; information not provided) and residential mobility (relocated to a different residential address since previous assessment: yes vs. no). Using a hearing aid (yes vs. no) was included as a covariate because hearing problems are a risk factor for dementia^36^ but not a potential mediator of the associations between cognitive states and the neighborhood attributes examined in this study.^37^

#### 2.3.3 Attributes of the neighborhood environment (exposures)

Detailed information on the spatial indicators, including data sources used to compute them and the Sydney MAS assessments (waves) they were linked to, are provided in the supporting information (pp. 1–3; Table S1). Here, we provide a brief overview.

For each study wave, we geocoded participants’ residential addresses at the building level and, in line with current recommendations, created 1 km radius road network buffers around them representing neighborhoods or areas within walking distance.^38^, ^39^, ^40^, ^41^, ^42^ Four categories of spatial indicators were computed for each residential buffer. These were area‐level socio‐economic status (SES), built environment features, natural environment features, and ambient air pollution.

Area‐level SES was treated as an environmental covariate or confounder in the regression models. It was operationalized as the weighted average of the Index of Relative Socio‐Economic Advantage and Disadvantage (IRSAD) provided by the Australian Bureau of Statistics.^43^ The neighborhood built environment was characterized by four spatial indicators: population density (persons of all ages/km^2^), street intersection density (intersections/km^2^), number of public transport points (sum of train stations, light rail stations, tram stops, bus stations/stops, ferry terminals, and taxi stands), and percentage of commercial land in the residential buffer. Three spatial indicators were used to describe the natural environment within a residential buffer. These were the percentages of parkland, blue space surface (water bodies), and tree canopy cover. Ambient air pollution was characterized by annual average concentrations of nitrogen dioxide (NO_2_) and PM_2.5_. These estimates were computed using national‐scale satellite‐based land‐use regression (LUR) models for Australia, gridded at ≈ 100 × ≈ 100 m, for each calendar year between 2005 and 2019, inclusive.^44^, ^45^ For analytical purposes, we used the original values of the spatial indicators as well as computed cumulative exposures for each wave representing the average values of a specific spatial indicator from baseline to that specific wave.^46^

### 2.4 Statistical analyses

Descriptive statistics were computed for all variables and study waves. Multi‐state Markov models with continuous time^47^ (package “msm” version 1.8.2 in R 4.3.3) were used to estimate the associations between neighborhood environmental attributes and the risk of two sets of transition states. The first set included data from all seven waves of the study and examined transitions from no dementia to dementia (Figure 2A). The second set included data from the first four waves of the study and examined transitions from normal cognition to MCI, from MCI to normal cognition, from MCI to dementia, and from normal cognition to dementia (Figure 2B). Death was modeled as an additional state rather than censored data (Figure 2A and B) and related findings are reported as supplementary analyses.

A first set of multi‐state models examined the overall associations of each environmental attribute with transition states adjusted for socio‐demographic factors, health conditions, and environmental confounders (overall effect model). A second set of models estimated the independent associations of each environmental attribute with transition states adjusted for all confounders as well as other environmental attributes that may act as mediators (independent effect models). Both original and cumulative values of environmental factors were examined in separate models, the former representing shorter term environmental exposures at the time of assessment and the latter representing longer term exposures from baseline to the time of assessment.

The inclusion of variables in specific multi‐state models were determined using directed acyclic graphs (DAGs; Figure S2 in supporting information) informed by the authors’ expert knowledge as detailed in Table S2 in supporting information. Models were inverse probability weighted to account for attrition bias. Inverse probability weights were defined as the inverse of the conditional probability of remaining in the study given participants’ characteristics at baseline and assessments (waves) prior to dropping out of the study (supporting information pp. 8–9; Table S3). Multicollinearity was assessed using variance inflation factor values of variables included in the models. Analyses were conducted in R 4.3.3.^48^ Sensitivity analyses, based on replacing unclassified cognitive states due to insufficient data with best possible cognitive state (optimistic) or worst possible cognitive state (pessimistic), were carried out to ensure they did not affect the findings (supporting information pp. 22–27).

## 3 RESULTS

Table 1 reports key socio‐demographic and health‐related characteristics of the participants assessed at each wave. The sample had an average age of 78.3 years at baseline and consisted of slightly more women than men, with the sex imbalance being more pronounced in later waves. Most participants were from an English‐speaking background, retired or unemployed at baseline, and living alone in the community. The percentage of participants moving to a different residential address between waves increased across time and ranged from 9.2% in Wave 2 to 15% in Wave 7. Approximately 20% of the sample had hearing aids at baseline, with the percentage increasing to 27.8% in Wave 7. The percentage of participants with a dementia diagnosis also gradually increased at each wave from 0% at baseline to > 35% at Wave 7. No such temporal pattern was observed for MCI cases (Table 1). From Wave 1 to 7, 264 transitions from no dementia to dementia were recorded. From Wave 1 to 4, there were 248 transitions from normal cognition to MCI and 173 from MCI to normal cognition. Furthermore, 65 participants transitioned from MCI to dementia and 17 transitioned from normal cognition to dementia.

**TABLE 1:** Participants’ socio‐demographic and health‐related characteristics by study wave.

Characteristics	Wave 1 (*n* = 1036)	Wave 2 (*n* = 888)	Wave 3 (*n* = 791)	Wave 4 (*n* = 707)	Wave 5 (*n* = 573)	Wave 6 (*n* = 475)	Wave 7 (*n* = 313)
Sex, female, %	55.1	54.1	53.5	55.2	56.7	59.2	63.3
Age, years, M ± SD	78.3 ± 4.8	80.0 ± 4.8	81.8 ± 4.7	83.6 ± 4.6	85.3 ± 4.5	87.2 ± 4.4	88.9 ± 3.9
Years of education (baseline)	11.6 ± 3.5	11.7 ± 3.5	11.7 ± 3.5	11.8 ± 3.5	11.8 ± 3.4	11.7 ± 3.4	12.0 ± 3.4
Tertiary education (baseline), %	30.0	30.3	30.7	31.5	32.3	31.2	32.6
Employed (baseline), %	11.8	12.8	13.5	13.9	15.2	15.2	16.9
Living arrangements, %							
Living in the community alone	46.8	47.0	46.9	45.0	43.3	37.9	36.4
Living in the community with partner	38.7	38.3	36.9	33.4	31.4	29.1	22.0
Other living arrangements	14.5	13.6	13.3	17.8	19.5	19.2	16.6
Information not provided	0.0	1.1	2.9	3.8	5.8	13.9	24.9
English‐speaking background, %	84.2	83.8	84.1	85.0	85.5	85.7	87.9
Hearing aid, yes, %	20.5	17.9	20.0	24.6	24.8	26.5	27.8
Residential relocation, yes, %	0.0	9.2	10.0	11.0	11.2	12.8	15.0
Cognitive state—classification 1, %							
No dementia	98.5	94.0	90.8	88.5	84.3	71.2	63.6
Dementia	0.0	2.7	6.7	11.5	14.5	26.1	35.1
Unclassifiable	1.5	3.3	2.5	0.0	1.2	2.7	1.3
Cognitive state—classification 2, %							
Normal cognition	53.3	49.7	53.1	50.5	–	–	–
Mild cognitive impairment	30.9	27.8	21.9	34.5	–	–	–
Dementia	0.0	2.7	6.7	11.5	–	–	–
Unclassifiable	15.8	19.8	18.3	3.5	–	–	–

*Note*: *n*, number of participants; M, mean; SD, standard deviation. Tertiary education refers to any formal education after high school.

Descriptive statistics of attributes of the neighborhood environment by study wave are reported in Table 2 for participants assessed at the specific waves. The environmental characteristics of participants who died between study waves were deemed to be the same as those at the last study wave they participated in. In general, participants resided in high SES areas with an average IRSAD score > 1000 (denoting average SES). Residential buffers (neighborhoods) had a higher percentage of tree canopy and parkland cover (≈ 20% and 12%, respectively) and smaller percentages of blue space (≈ 9%) and commercial land (≈ 4%–7%). Annual average concentrations of air pollutants were low and below the Australian national standards for both PM_2.5_ (8 µg/m^3^) and NO_2_ (15 ppb).^49^ Between‐participant variability in exposures was substantial (defined as a coefficient of variation > 50%) for percentage of commercial land, parkland, and blue space. The remaining attributes showed moderate (20%–50%: street intersection density; transit points; tree canopy cover) to low variability (5%–19%: population density; PM_2.5_; NO_2_) across participants.

**TABLE 2:** Attributes of the neighborhood environment by study wave (M ± SD).

Attribute	Value	Wave 1 (*n* = 1036)	Wave 2 (*n* = 888)	Wave 3 (*n* = 791)	Wave 4 (*n* = 707)	Wave 5 (*n* = 573)	Wave 6 (*n* = 475)	Wave 7 (*n* = 313)
Population density (people/km^2^)	Original	6571 ± 1231	6501 ± 1372	6692 ± 1614	6813 ± 1772	7004 ± 1976	7181 ± 2108	7141 ± 2059
	Cumulative	6571 ± 1231	6538 ± 1278	6549 ± 1351	6593 ± 1396	6671 ± 1425	6709 ± 1484	6702 ± 1535
Street intersection density (intersections/km^2^)	Original	95.3 ± 23.6	94.5 ± 24.8	79.6 ± 15.7	64.7 ± 11.4	80.2 ± 15.5	96.0 ± 24.5	96.5 ± 24.3
	Cumulative	95.3 ± 23.6	94.9 ± 23.8	89.2 ± 20.8	82.8 ± 16.8	82.0 ± 15.8	83.9 ± 14.4	84.9 ± 17.5
Transit points (count)	Original	41.2 ± 13.9	40.8 ± 14.1	40.8 ± 14.1	40.5 ± 14.2	39.8 ± 14.2	39.9 ± 13.6	39.8 ± 13.2
	Cumulative	41.2 ± 13.9	41.0 ± 13.9	40.8 ± 13.7	40.6 ± 13.7	40.3 ± 13.3	40.0 ± 12.5	39.2 ± 12.6
Commercial land (%)	Original	6.7 ± 5.3	6.9 ± 6.3	6.7 ± 6.3	6.4 ± 6.3	5.0 ± 5.5	3.7 ± 5.0	3.7 ± 4.7
	Cumulative	6.7 ± 5.3	6.8 ± 5.6	6.7 ± 5.7	6.6 ± 5.7	6.2 ± 5.5	5.8 ± 5.2	5.3 ± 5.2
Parkland (%)	Original	12.2 ± 7.3	12.4 ± 7.7	12.3 ± 8.0	12.3 ± 8.2	12.2 ± 8.7	11.9 ± 8.8	12.0 ± 8.8
	Cumulative	12.2 ± 7.3	12.3 ± 7.4	12.2 ± 7.4	12.0 ± 7.2	12.1 ± 7.4	12.1 ± 7.4	12.2 ± 7.8
Tree canopy cover (%)	Original	20.6 ± 4.1	20.7 ± 4.5	20.6 ± 4.8	20.8 ± 5.4	20.8 ± 5.8	21.1 ± 5.9	21.8 ± 5.9
	Cumulative	20.6 ± 4.1	20.7 ± 4.3	20.5 ± 4.4	20.4 ± 4.5	20.6 ± 4.6	20.5 ± 4.8	20.9 ± 5.0
Blue space (%)	Original	8.6 ± 16.2	8.9 ± 15.3	8.8 ± 14.9	8.9 ± 15.0	9.0 ± 14.7	9.0 ± 14.6	9.1 ± 15.2
	Cumulative	8.6 ± 16.2	8.8 ± 15.2	8.6 ± 14.7	8.6 ± 14.5	8.6 ± 14.3	8.7 ± 14.7	9.1 ± 14.3
PM_2.5_ (µg/m^3^)	Original	7.1 ± 0.2	7.1 ± 0.3	7.0 ± 0.4	6.8 ± 0.4	7.0 ± 0.5	7.0 ± 0.5	7.0 ± 0.4
	Cumulative	7.1 ± 0.2	7.1 ± 0.3	7.0 ± 0.4	6.9 ± 0.5	6.9 ± 0.5	6.9 ± 0.5	6.9 ± 0.5
NO_2_ (ppb)	Original	14.9 ± 1.2	14.8 ± 1.3	14.2 ± 1.4	13.5 ± 1.5	13.0 ± 1.6	13.1 ± 1.5	13.1 ± 1.4
	Cumulative	14.9 ± 1.2	14.9 ± 1.2	14.6 ± 1.4	14.2 ± 1.5	14.0 ± 1.4	13.7 ± 1.4	13.6 ± 1.4
Area‐level IRSAD	Original	1147 ± 27.9	1144 ± 41.5	1085 ± 66.3	1031 ± 102.8	1043 ± 114.9	1053 ± 118.0	1055 ± 110.9
	Cumulative	1147 ± 27.9	1146 ± 32.3	1119 ± 63.7	1092 ± 73.3	1081 ± 77.9	1070 ± 83.7	1067 ± 90.7

*Notes*: Original = estimated exposure values at a specific wave; Cumulative = average estimated exposure from baseline to the specific wave. Only participant that were assessed at a specific study wave are included. Participants who died between study waves were assumed to have the same environmental exposures at the time of death as those of the last wave in which they participated in the study.

Abbreviations: IRSAD, Index of Relative Social Advantage and Disadvantage; M, mean; NO_2_, nitrogen dioxide; PM_2.5_, particulate matter with a diameter of 2.5 µm (micrometers) or smaller; ppb, parts per billion; SD, standard deviation.

### 3.1 Neighborhood environmental attributes and transitions to cognitive states

Given that the analyses of transitions to various cognitive states were based on different sample sizes and number of waves, we tabulated the findings by pairs of cognitive states with equal sample sizes and number of waves. Overall‐ and independent‐effect estimates of associations between neighborhood environmental attributes and transition to dementia across the follow‐up period are reported in Table 3, while Tables 4 and 5 show associations of environmental attributes with transitions between cognitive states using the classifications of normal cognition, MCI, and dementia over 6 years of follow‐up. The associations between neighborhood environmental attributes, informed by models described in the supporting information (p. 10; Table S4), are reported in Figures S3–S20, and those of environmental attributes with transitions to death in Tables S5 and S6. Variance inflation factors did not exceed 2.40, evidencing no potential multicollinearity problems.^50^ It is important to note that, in interpreting these associations, we assume that they are stable across time. Also, we cannot discern the extent to which they represent between‐person effects (i.e., differences between individuals) or within‐person effects (changes within individuals).^51^

**TABLE 3:** Neighborhood environmental attributes as correlates of transitions from no dementia to dementia: Overall‐ and independent‐effect models (*N* = 1036 followed for up to 13.7 years).

Neighborhood environmental attribute		Overall‐effect models	Independent‐effect models
	Spatial indicator	HR (95% CI)	HR (95% CI)
Population density (100 persons/km^2^)	Original	1.001 (0.994, 1.009)	**0.995 (0.990, 0.999)**
	Cumulative	1.002 (0.993, 1.012)	0.998 (0.990, 1.006)
Street intersection density (intersections/km^2^)	Original	1.000 (0.993, 1.006)	0.996 (0.988, 1.004)
	Cumulative	0.998 (0.991, 1.006)	1.001 (0.996, 1.005)
Transit points (count)	Original	**0.992 (0.984, 0.999)**	**0.986 (0.973, 0.998)**
	Cumulative	0.992 (0.981, 1.003)	**0.987 (0.975, 0.999)**
Commercial land (%)	Original	**0.935 (0.893, 0.979)**	0.992 (0.966, 1.018)
	Cumulative	**0.980 (0.962, 0.998)**	0.985 (0.960, 1.012)
Parkland (%)	Original	**0.990 (0.983, 0.997)**	1.002 (0.982, 1.022)
	Cumulative	0.997 (0.979, 1.014)	0.990 (0.969, 1.011)
Tree canopy cover (%)	Original	**0.961 (0.935, 0.987)**	**0.955 (0.929, 0.983)**
	Cumulative	**0.967 (0.936, 0.998)**	**0.973 (0.950, 0.997)**
Blue space (%)	Original	0.997 (0.989, 1.008)	**0.987 (0.976, 0.999)**
	Cumulative	1.001 (0.991, 1.011)	0.995 (0.982, 1.008)
NO_2_ (ppb)	Original	**0.898 (0.809, 0.995)**	**0.855 (0.762, 0.959)**
	Cumulative	1.020 (0.901, 1.154)	0.917 (0.787, 1.067)
PM_2.5_ (µg/m^3^)	Original	1.011 (0.720, 1.420)	1.125 (0.802, 1.578)
	Cumulative	**1.475 (1.046, 2.079)**	**1.561 (1.046, 2.329)**

*Notes*: Estimates adjusted for confounders. Inverse probability weights were used to account for attrition bias. Statistically significant effects (*P* < 0.05) are in bold. Eighty‐one out of 4154 observations (1.9%) had insufficient cognitive state data. Two hundred sixty‐our transitions to dementia were recorded. Environmental correlates of transitions from no dementia to death and from dementia to death can be found in the supporting information (Table S4) as they were not the focus of the study. Overall‐effect estimates were adjusted for confounders, while independent‐effect estimates were also adjusted for potential environmental mediators.

Abbreviations: CI, confidence interval; HR, hazard ratio; NO_2_, nitrogen dioxide; PM_2.5_, particulate matter with a diameter of 2.5 µm (micrometers) or smaller; ppb, parts per billion.

**TABLE 4:** Neighborhood environmental attributes as correlates of transition (progression) to worse cognitive states: Overall‐ and independent‐effect models (*N *= 1036 followed up for ≈ 6 years).

		Overall‐effect models	Independent‐effect models	Overall‐effect models	Independent‐effect models	Overall‐effect models	Independent‐effect models
Neighborhood environmental attribute	Spatial indicator	Normal cognition to MCI	Normal cognition to MCI	Normal cognition to dementia	Normal cognition to dementia	MCI to dementia	MCI to dementia
		HR (95% CI)	HR (95% CI)	HR (95% CI)	HR (95% CI)	HR (95% CI)	HR (95% CI)
Population density (100 persons/km^2^)	Original	**0.995 (0.989, 0.999)**	1.000 (0.988, 1.013)	1.009 (0.953, 1.068)	**0.983 (0.971, 0.995)**	0.997 (0.981, 1.013)	1.015 (0.996, 1.034)
	Cumulative	**0.994 (0.988, 0.999)**	0.999 (0.985, 1.013)	1.020 (0.974, 1.068)	**0.969 (0.953, 0.987)**	1.000 (0.987, 1.013)	1.015 (0.992, 1.038)
Street intersection density (intersections/km^2^)	Original	**0.995 (0.991, 0.998)**	**0.992 (0.984, 0.999)**	**1.067 (1.030, 1.107)**	1.060 (0.987, 1.138)	**0.993 (0.987, 0.999)**	1.000 (0.987, 1.014)
	Cumulative	0.997 (0.991, 1.003)	**0.994 (0.988, 0.999)**	**1.054 (1,005, 1.106)**	**0.946 (0.897, 0.996)**	**0.992 (0.985, 0.998)**	1.000 (0.982, 1.018)
Transit points (count)	Original	0.991 (0.965, 1.018)	1.005 (0.991, 1.020)	1.014 (0.726, 1.416)	1.126 (0.930, 1.363)	**0.967 (0.949, 0.985)**	**0.974 (0.950, 0.998)**
	Cumulative	0.988 (0.961, 1.015)	1.010 (0.995, 1.025)	1.057 (0.836, 1.337)	1.160 (0.967, 1.392)	**0.969 (0.950, 0.988)**	**0.961 (0.935, 0.988)**
Commercial land (%)	Original	**0.971 (0.944, 0.998)**	**0.961 (0.924, 0.999)**	1.078 (0.991, 1.174)	**0.743 (0.624, 0.934)**	**0.974 (0.951, 0.998)**	0.974 (0.914, 1.037)
	Cumulative	**0.972 (0.946, 0.999)**	**0.959 (0.922, 0.998)**	**1.164 (1.036, 1.310)**	**0.745 (0.581, 0.955)**	**0.976 (0.957, 0.996)**	0.973 (0.912, 1.037)
Parkland (%)	Original	1.016 (0.997, 1.036)	1.012 (0.991, 1.034)	0.943 (0.800, 1.111)	0.992 (0.833, 1.181)	1.013 (0.986, 1.042)	0.997 (0.966, 1.030)
	Cumulative	**1.023 (1.002, 1.044)**	1.016 (0.991, 1.040)	1.084 (0.948, 1.240)	1.055 (0.871, 1.278)	1.009 (0.982, 1.036)	0.991 (0.953, 1.030)
Tree canopy cover (%)	Original	**0.975 (0.953, 0.998)**	**0.966 (0.934, 0.998)**	0.888 (0.676, 1.166)	0.852 (0.611, 1.189)	**0.951 (0.908, 0.999)**	0.966 (0.902, 1.035)
	Cumulative	**0.970 (0.943, 0.998)**	0.979 (0.932, 1.028)	0.887 (0.533, 1.476)	0.946 (0.619, 1.444)	**0.945 (0.895, 0.998)**	0.953 (0.878, 1.035)
Blue space (%)	Original	1.002 (0.992, 1.012)	1.003 (0.990, 1.017)	0.869 (0.669, 1.130)	0.933 (0.706, 1.233)	1.002 (0.985, 1.019)	0.995 (0.975, 1.016)
	Cumulative	1.002 (0.992, 1.013)	1.008 (0.994, 1.023)	0.859 (0.541, 1.364)	0.933 (0.773, 1.126)	1.004 (0.988, 1.019)	0.993 (0.972, 1.015)
NO_2_ (ppb)	Original	**0.904 (0.819, 0.997)**	1.051 (0.878, 1.257)	**2.366 (1.336, 4.189)**	**4.724 (1.118, 19.962)**	0.990 (0.831, 1.178)	1.022 (0.767, 1.363)
	Cumulative	1.006 (0.869, 1.163)	1.065 (0.885, 1.281)	**4.634 (1.107, 19.400)**	3.493 (0.684, 17.849)	1.173 (0.917, 1.501)	1.008 (0.729, 1.393)
PM_2.5_ (µg/m^3^)	Original	0.933 (0.538, 1.612)	1.015 (0.876, 1.177)	1.461 (0.973, 2.195)	2.513 (0.785, 8.045)	1.470 (0.616, 3.504)	1.025 (0.895, 1.155)
	Cumulative	0.938 (0.536, 1.640)	1.078 (0.942, 1.235)	1.494 (0.882, 2.529)	2.191 (0.685, 7.007)	2.973 (0.893, 9.901)	0.991 (0.737, 1.332)

*Notes*: Estimates adjusted for confounders. Inverse probability weights were used to account for attrition bias. Statistically significant effects (*p* < 0.05) are in bold. Four hundred seventy‐four out of 2530 observations (18.7%) with insufficient cognitive state data. Two hundred forty‐eight and 17 transitions from normal cognition to, respectively, MCI and dementia were recorded. Sixty‐five transitions from MCI to dementia were recorded. Environmental correlates of transitions to death can be found in the supporting information (Table S5) as they were not the focus of the study, while those of transitions from MCI to normal cognition are in Table 5. Overall‐effect estimates were adjusted for confounders, while independent‐effect estimates were also adjusted for potential environmental mediators.

Abbreviations: CI, confidence interval; HR, hazard ratio; MCI, mild cognitive impairment; NO_2_, nitrogen dioxide; PM_2.5_, particulate matter with a diameter of 2.5 µm (micrometers) or smaller; ppb, parts per billion.

**TABLE 5:** Neighborhood environmental attributes as correlates of reversal from mild cognitive impairment to normal cognition: Overall‐ and independent‐effect models (*N* = 1036 followed up for ≈ 6 years).

Neighborhood environmental attribute		Overall‐effect models	Independent‐effect models
	Spatial indicator	HR (95% CI)	HR (95% CI)
Population density (100 persons/km^2^)	Original	1.003 (0.992, 1.014)	1.003 (0.987, 1.018)
	Cumulative	0.997 (0.988, 1.006)	1.003 (0.986, 1.020)
Street intersection density (intersections/km^2^)	Original	0.998 (0.991, 1.006)	0.998 (0.986, 1.010)
	Cumulative	0.998 (0.991, 1.005)	0.999 (0.989, 1.008)
Transit points (count)	Original	1.001 (0.988, 1.014)	**1.018 (1.000, 1.036)**
	Cumulative	1.002 (0.989, 1.015)	**1.021 (1.003, 1.039)**
Commercial land (%)	Original	0.980 (0.952, 1.008)	0.961 (0.918, 1.005)
	Cumulative	0.978 (0.950, 1.007)	0.964 (0.921, 1.009)
Parkland (%)	Original	**1.018 (1.001, 1.036)**	1.011 (0.988, 1.035)
	Cumulative	**1.019 (1.000, 1.039)**	1.014 (0.988, 1.039)
Tree canopy cover (%)	Original	0.970 (0.928, 1.015)	0.969 (0.921, 1.020)
	Cumulative	0.969 (0.922, 1.018)	0.988 (0.929, 1.051)
Blue space (%)	Original	1.009 (0.997, 1.021)	**1.016 (1.001, 1.032)**
	Cumulative	1.009 (0.997, 1.022)	**1.020 (1.003, 1.036)**
NO_2_ (ppb)	Original	0.943 (0.835, 1.065)	1.109 (0.884, 1.391)
	Cumulative	0.941 (0.785. 1.147)	1.073 (0.849. 1.355)
PM_2.5_ (µg/m^3^)	Original	0.947 (0.511, 1.758)	0.948 (0.777, 1.156)
	Cumulative	1.016 (0.525, 1.963)	1.111 (0.929, 1.330)

*Notes*. Estimates adjusted for confounders. Inverse probability weights were used to account for attrition bias. Statistically significant effects (*P* < 0.05) are in bold. Four hundred seventy‐four out of 2530 observations (18.7%) with insufficient cognitive state data. One hundred seventy‐three transitions from MCI to normal cognition were recorded. Overall‐effect estimates were adjusted for confounders, while independent‐effect estimates were also adjusted for potential environmental mediators.

Abbreviations: CI, confidence interval; HR, hazard ratio; NO_2_, nitrogen dioxide; PM_2.5_, particulate matter with a diameter of 2.5 µm (micrometers) or smaller; ppb, parts per billion.

Although the results are tabulated by pairs of cognitive states, we summarize and interpret the findings by environmental attribute to provide a holistic picture of their impacts on older adults’ cognitive health. Residents of neighborhoods with higher population density were less likely to transition from no dementia to dementia (Table 3) and from normal cognition to dementia (Table 4), but only after adjustment for other environmental variables acting as potential intermediate factors (mediators) on the pathway from population density and cognitive state (e.g., air pollutants; independent‐effect models). They were also less likely to transition from normal cognition to MCI, but only when potential environmental mediators were unaccounted for (Table 4; overall‐effect models). Higher street intersection density was associated with a lower likelihood of transitioning from normal cognition to MCI, especially in the independent‐effect models and a lower likelihood of transitioning from MCI to dementia in the overall‐effect models (Table 4). Conversely, higher street intersection density was associated with a higher likelihood of transitioning from normal cognition to dementia between study waves in the overall‐effect model (Table 4).

Having more transit points in the neighborhood was associated with better cognitive outcomes, including a lower likelihood of transitioning from no dementia to dementia (Table 3) and from MCI to dementia (Table 4), and a higher likelihood of reversal from MCI to normal cognition (Table 5). Of note is that these associations were stronger, or emerged, after adjustment for potential environmental mediators (independent‐effect models).

Residents of neighborhoods with more commercial land had a lower probability of transitioning from no dementia to dementia (Table 3), from normal cognition to MCI, from MCI to dementia, and from normal cognition to dementia (Table 4). However, except for transitions from normal cognition to MCI, these associations depended on whether the estimates were adjusted for environmental mediators. Specifically, overall‐effect associations were statistically significant for transitions from no dementia and MCI to dementia, while independent‐effect associations were significant for transitions from normal cognition to dementia. Having more parkland in the neighborhood was associated with a smaller likelihood of transitioning from no dementia to dementia (Table 3) and a greater likelihood of reverting from MCI to normal cognition (Table 5), but only in the overall‐effect models. In contrast, the same environmental attribute tended to be associated with a higher likelihood of transitioning from normal cognition to MCI (Table 4).

Tree canopy cover was consistently predictive of a lower likelihood of transitions from no dementia to dementia (Table 3) and from normal cognition to MCI (Table 4). Similar, albeit more uncertain, effects were observed in relation to transitions from MCI to dementia (Table 4). Participants with a higher percentage of blue spaces were less likely to transition from no dementia to dementia (Table 3) and more likely to revert from MCI to normal cognition (Table 5), but only in the independent‐effect models.

Higher estimates of average annual concentrations of NO_2_ at a specific wave were associated with a lower likelihood of transitioning from no dementia to dementia (Table 3) and from normal cognition to MCI (Table 4). However, the opposite was observed with respect to transitions from normal cognition to dementia (Table 4). It is also noteworthy that estimates of cumulative exposures were, instead, leaning toward opposite (harmful) effects. Finally, estimates of cumulative exposure to PM_2.5_ were associated with a higher likelihood of transitioning from no dementia to dementia (Table 3). No other marked differences in associations between cumulative and shorter term (i.e., original) spatial indicators of other environmental attributes with transitions to cognitive states were observed except for street intersection density in relation to transitions from normal cognition to dementia (Table 4).

Sensitivity analyses with unclassifiable cognitive state data replaced by the best possible diagnosis (Scenario 1—optimistic) and worse possible diagnosis (Scenario 2—pessimistic) resulted in similar patterns of associations, albeit attenuated, with a few exceptions (Tables S7–S11 in supporting information).

## 4 DISCUSSION

Using data from a population‐based cohort study of older Australians, we examined a comprehensive range of interrelated neighborhood environmental attributes as correlates of transitions to MCI and dementia and reversal from MCI to normal cognition. In doing so, we examined shorter and longer‐term (cumulative) environmental exposures, and estimated the overall (i.e., via other environmental attributes) as well as unique (i.e., independent) contributions of each environmental attribute. Overall, more dense neighborhoods, with more interconnected streets, better access to public transport and commercial services, and more tree canopy cover were associated with a lower likelihood of transitioning to a worse cognitive state, while higher cumulative exposure to PM_2.5_ was associated with a higher likelihood of transitioning to dementia. Less compelling or conflicting evidence was found in relation to parkland, blue spaces (water bodies), and annual average exposure to NO_2_. Only access to public transport, parkland, and blue spaces were associated with a higher likelihood of reversals from an MCI diagnosis to a normal cognition diagnosis. We discuss findings by category of environmental attribute, starting from the built environment.

Dense, walkable neighborhoods are deemed to be beneficial to older adults’ cognitive health by facilitating engagement in educational, physical, and social activities.^9^, ^16^, ^37^ Living in such neighborhoods has been linked to better cognitive^8^, ^9^, ^16^ as well as brain health.^9^, ^13^ Hence, findings suggestive of protective effects of population density, street connectivity, and access to commercial services and public transport against cognitive decline were expected,^8^, ^9^, ^16^ especially for estimates adjusted for ambient air pollution, which is a by‐product of densification as well as a health hazard.^12^ Indeed, 62% of the association estimates (hazard ratios) were stronger and in the expected direction in the independent effect rather than overall effect models (Tables 3, 4, 5). The fact that the direction of the associations of street intersection density and commercial land with the probability of transition from normal cognition to dementia reversed (in the expected direction) after adjustment air pollution (and other environmental mediators) highlights the importance of these environmental features and considering both overall and independent effects of environmental attributes on cognitive states, which only a few recent studies have done.^9^

Results from a cohort of young‐old Australians (aged 60–64 years at baseline) found evidence of potential protective effects of similar environmental attributes against incident MCI.^19^ However, the same study reported population density, access to public transport, and commercial land to be associated with a lower likelihood of reversal from MCI to no MCI (i.e., cognitive improvement), which was not the case in the present investigation. The previous study of young‐old adults might have had too few MCI/dementia cases in high walkable environments to provide sufficient variability in reversal outcomes. It is interesting that we found access to public transport to be the only built environment attribute associated with a higher probability of reversal from MCI to no MCI. Whether the lack of environmental correlates of MCI reversal is due to diagnostic inaccuracy or limited effects of the environment on cognitive improvement after the onset of MCI is yet to be established.

Access to the natural environment, such as parks, treed avenues, or coastal waters, may provide enhanced opportunities for physical and social activities,^52^, ^53^ restore voluntary attention,^54^ and reduce stress^55^ and air pollution,^56^ all of which benefit cognitive health.^37^ In line with these assumptions, the present study found consistent evidence of a negative association between the amount of tree canopy in a neighborhood and the likelihood of transitioning to a worse cognitive state. In contrast, the percentage of parkland in the neighborhood showed a potentially beneficial effect by lowering the likelihood of transitions from no dementia to dementia and increasing the likelihood of reversal from MCI to normal cognition, but also a potentially detrimental effect in relation to transitions from normal cognition to MCI. Unlike tree canopy cover, parkland can represent extremely diverse environments (e.g., aesthetically pleasing and treed urban parks; sports fields; vacant unkept grassland), some of which may facilitate and other which may deter spending time outdoors and participating in activities. Therefore, it is plausible that this particular spatial indicator of the neighborhood natural environment may produce mixed findings^9^ and may indeed need to be replaced by more precise indicators (tree canopy cover; park presence and quality) in future studies. In this regard, the published evidence on the relationship between green spaces and cognitive health in older adults is much more consistent for tree canopy and woodland than for more generic indicators such as parkland or the normalized difference vegetation index (NDVI).^9^, ^57^ Apart from measurement‐related reasons, this has been attributed to tree canopies lowering air pollution and providing shade and temperature regulation encouraging older adults to be more active in the community.^9^

Access to blue spaces (in this case, the ocean, and, to a lesser extent, inland water bodies) was predictive of a lower likelihood of transitioning from no dementia to dementia and higher likelihood of reversal from MCI to normal cognition. These associations emerged only after accounting for other environmental attributes. The evidence of the potential effects of exposure to blue spaces on cognitive health in older adults is sparse.^58^, ^59^, ^60^ We are aware of only one cohort study that examined presence of blue spaces in the neighborhood as a correlate of transitions from no MCI to MCI and vice versa in an Australian cohort of young‐old adults.^19^ The study found evidence of a potential protective effect against incident MCI. The present study adds to the evidence suggesting that living near coastal waters may also help to delay the onset of dementia.

This study also examined the contribution of annual averages of NO_2_ and PM_2.5_ to transitions to cognitive states. We expected both pollutants would display consistent associations indicative of detrimental effects on cognition because there are plausible and demonstrated biological mechanisms linking them to neurodegeneration.^36^, ^61^, ^62^ However, findings were inconsistent. NO_2_ was associated with worse cognitive outcomes only in relation to transitions from normal cognition to dementia in a small group of participants, while PM_2.5_ was predictive of a higher likelihood of transitioning from no dementia to dementia only when using estimates of cumulative exposure. In addition, NO_2_ showed negative rather than positive associations with the likelihoods of transitions from no dementia to dementia and from normal cognition to MCI. The generally low levels of, and limited variability in, annual average concentrations of these air pollutants may have contributed to the general lack of associations. The person‐level standard deviation for PM_2.5_ ranged from 0.2 to 0.5 μ/m^3^ and that of NO_2_ was 1.2 to 1.6 ppb, while previous studies conducted in Australia reported standard deviations of 1.4 to 1.7 μ/m^3^ and 1.8 to 2.1 ppb for PM_2.5_ and NO_2_, respectively.^19^, ^21^ Also, the mean annual average concentrations of PM_2.5_ and NO_2_ observed in this study were below the Australian national air quality standards^49^ and > 50% lower than those reported in other geographical locations.^63^, ^64^, ^65^ It is possible that the concentrations of air pollutants might have been too low to significantly contribute to cognitive decline. In general, and mirroring the findings from our study, PM_2.5_ has been more consistently associated with incident dementia than NO_2_.^20^ NO_2_ being one of the main traffic‐related air pollutants^66^ may be also an indicator of neighborhood vibrancy and social activities,^21^, ^60^ which may somewhat counteract its harmful effects on cardiometabolic health and other biological pathways contributing to dementia.^61^

Apart from being the first study to examine a wide range of key neighborhood characteristics as correlates of transition to dementia and other cognitive states, this study has additional strengths. These include a long follow‐up time (up to 13.7 years) with up to seven biennial assessments of cognitive states, consensus diagnoses by an expert panel, repeated measures of neighborhood attributes, considering causal relationships between environmental variables, and modeling death as a competing outcome rather than censored state. Limitations are having environmental reference data for only up to three time points across the study duration introducing error in exposure estimates and establishment of temporal causality; having coarse measures of commercial land and parkland possibly leading to attenuated associations; not having information on the proportion of the day participants typically spent in the neighborhood, earlier environmental exposures, length of residence in the neighborhood prior to the study, and residential self‐selection; participants being recruited from a limited number of high‐SES neighborhoods with low variability in ambient air pollution limiting the generalizability of the findings and possibly resulting in attenuated associations; the significant loss of participants over time; and limited cognitive assessments for Waves 5 to 7.

## 5 CONCLUSION

This study suggests that, in socially advantaged urban areas with relatively low levels of ambient air pollution, living in walkable, leafy neighborhoods near coastal waters and with good access to commercial services and public transport may delay cognitive decline and the onset of dementia in people aged ≥ 70 years. These findings have implications for the urban design of age‐friendly cities, the planning of retirement villages and age care facilities, and the identification of at‐risk groups of older adults requiring interventions to reduce the risk of cognitive decline. They also suggest that public health and policy initiatives should prioritize and implement evidence‐based environmental interventions. Further research in diverse populations and geographical contexts is needed to cross‐validate the findings.

## AUTHOR CONTRIBUTION

**Ester Cerin**: writing—review & editing, writing—original draft, supervision, project administration, methodology, investigation, funding acquisition, formal analysis, conceptualization. **Annabel P. Matison**: writing—review & editing, writing—original draft, validation, data curation, conceptualization. **Miguel A. Molina**: writing—review & editing, validation, supervision, software, methodology, investigation, data curation. **Ralf‐Dieter Schroers**: writing—review & editing, validation, software, methodology, investigation, data curation. **Wei Li**: writing—review & editing, validation, software, methodology, data curation. **Luke D. Knibbs**: writing—review & editing, methodology, funding acquisition, data curation. **Vibeke Sorensen Catts**: writing—review & editing, data curation, data management, project administration, conceptualization. **Yu‐Tzu Wu**: writing—review & editing, software, funding acquisition, conceptualization. **Maria V. Soloveva**: writing—review & editing, writing—original draft, project administration. **Kaarin J. Anstey**: writing—review & editing, funding acquisition, conceptualization. **Suzanne Mavoa**: writing—review & editing, supervision, funding acquisition. **Govinda Poudel**: writing—review & editing, writing—original draft. **Bin Jalaludin**: writing—review & editing, funding acquisition. **Nicole A. Kochan**: writing—review & editing, data acquisition and management, methodology. **Henry Brodaty**: writing—review & editing, data acquisition, supervision, methodology, investigation, funding acquisition, resources. **Perminder S. Sachdev**: writing—review & editing, data acquisition, supervision, methodology, investigation, funding acquisition, resources, conceptualization.

## CONFLICT OF INTEREST STATEMENT

Perminder S. Sachdev reports serving on the expert advisory committees of Biogen and Roche Australia in 2020 and 2021; and receiving speaker fees from Alkim Laboratories. Henry Brodaty is or has been an advisory board member or consultant to Biogen, Eisai, Eli Lilly, Medicines Australia, Roche, and Skin2Neuron. Other authors (Ester Cerin, Annabel P. Matison, Miguel A. Molina, Ralf‐Dieter Schroers, Wei Li, Luke D. Knibbs, Vibeke Sorensen Catts, Yu‐Tzu Wu, Maria V. Soloveva, Kaarin J. Anstey, Suzanne Mavoa, Govinda Poudel, Bin Jalaludin, and Nicole A. Kochan) declare no conflicts of interest. Author disclosures are available in the supporting information.

## CONSENT STATEMENT

All participants of this study provided informed consent.

## Supporting information



Supporting information

Supporting information
