# Supplementary material for: Neighborhood environments and transition to cognitive states: Sydney Memory and Ageing Study
Source: Alzheimers Dement. 2025 Aug 5;21(8):e70569. doi: 10.1002/alz.70569 (PMC12325892; doi:10.1002/alz.70569)
Supplement: Supplementary file 1 — Supporting information [file ALZ-21-e70569-s002.docx]

**Supplementary Material**

*Attributes of the neighbourhood environment (exposures) – detailed description*

For each study wave, we geocoded participants’ residential addresses at the building level and created 1-km-radius (standard) road network buffers around the participants’ geocoded residential addresses [1, 2] with ArcGIS v.10.6 network analyst [3]. These buffers were created using networks of paths and roads accessible to people. The decision to use a 1-km radius to create residential buffers representing a participant’s neighbourhood was based on the fact that this size of buffers has yielded stronger associations with health outcomes in older Australians compared to smaller (e.g., 500-m radius) or larger (e.g., 1.6-km radius) buffers [4, 5] and is the most common buffer size employed in international studies [1, 6, 7].

As the Sydney Memory and Ageing Study (Sydney MAS) collected data across seven waves, 411 participants relocated between waves and urban environments change across time, we created residential buffers for all 7 waves. Four categories of spatial indicators were computed for each residential buffer:

- area-level socio-economic status (SES) (a single indicator)
- built environment features (four indicators)
- natural environment features (three indicators)
- ambient air pollution (two indicators).

*Area-level SES* was treated as an environmental covariate or confounder in the regression models. It was operationalised as the weighted average of the Index of Relative Socio-Economic Advantage and Disadvantage (IRSAD) provided by the Australian Bureau of Statistics (ABS) (weighted by the proportion of the buffer area belonging to specific SA1s). This index is a general measure of advantage and disadvantage with high scores representing areas with a high income and a high proportion of people in skilled occupations [8]. The neighbourhood *built environment* was characterized by four spatial indicators: population density (persons/km^2^), street intersection density (≥3-arm intersections/km^2^), number of public transport points (sum of train stations, light rail stations, tram stops, bus stations/stops, ferry terminals and taxi stands) and percentage of commercial land in the residential buffer. Three spatial indicators were used to describe the *natural environment* within a residential buffer. These were the percentages of parkland, blue space surface (water bodies) and tree cover.

Ambient air pollution was characterised by two air pollutants: nitrogen dioxide (NO_2_) and fine particulate matter defined as particles that are 2.5 microns or less in diameter (PM_2.5_). We assigned annual average PM_2.5_ and NO_2_ concentrations using two national-scale satellite-based land-use regression (LUR) models for Australia, gridded at ~100 × ~100 m, for each calendar year during 2005-2018 inclusive (2005 was the full year of satellite NO_2_ observations). The LUR models captured an estimated 81% (RMSE: 1.4 ppb) of spatial variability in annual NO_2_, and an estimated 63% (RMSE: 1 μg/m^3^) of spatial variability in annual PM_2.5_ [9, 10]. Both models undergo ongoing checking, updates and testing to incorporate new predictors and monitor data as needed, as well as validation against databases of historical or independent monitoring sites (sites not used to develop the models) [9, 11]. The underlying resolution is determined by the spatial variation of the least granular predictor, and in practice is up to ~100 m in urban areas and up to ~500 m in rural areas [12]. The centroid of each ~100 m grid cell was used to estimate LUR predictions from the models, which included predictors on natural and anthropogenic features (including satellite estimates of NO_2_ and PM_2.5_) that have a plausible association (negative or positive) with PM_2.5_ and/or NO_2_, as measured by regulatory monitors using standard reference methods. The LUR estimates were linked to geocoded addresses based on the cell they were within.

Table S1 provides information on the data sources used to compute the spatial indicators and the Sydney MAS assessments (waves) they were linked to (e.g., population density derived from the 2006 Census database was linked to Sydney MAS data from Waves 1 and 2). Over 90% of Wave 1-2, Wave 3-4 and Wave 5-7 assessments were, respectively, conducted in 2006-2009, 2010-2013 and 2014-2020. For analytical purposes, apart from using the original values of the spatial indicators, we computed cumulative exposures for each wave representing the average values of a specific spatial indicator from baseline to that specific wave [13].

**Table S1.** Data sources used to compute spatial indicators of neighbourhood attributes for the Sydney Memory and Ageing Study (Sydney MAS)

| **Category of spatial indicator** | **Spatial indicator** | **Data sources** | **Year(s) of spatial data [Sydney MAS study waves (W) linked to data]** |
| --- | --- | --- | --- |
| Area-level socio-economic status | Index of Relative Socio-Economic Advantage and Disadvantage | Australian Bureau of Statistics. Census of Population and Housing: Socio-Economic Indexes for Areas (SEIFA), Australia. | 2006 [W1-W2]  2011 [W3-W4]  2016 [W5-W7] |
| Built environment | Population density | Australian Bureau of Statistics. Census of Population and Housing: Mesh Block Counts. | 2006 [W1-W2]  2011 [W3-W4]  2016 [W5-W7] |
|  | Street intersection density | PSMA Australia Limited. Transport and Topography. Street Line. Only walkable roads were used. | 2012 [W1-W4]  2018 [W5-W7]  Note: 2006 dataset of poor quality and, hence, not used |
|  | Number of public transport points | PMSA Australia Limited. Train Routes.  Public Transport Authority (New South Wales) | 2012 [W1-W4]  2018 [W5-W7]  2013 [W1-W4]  2017 [W5-W7] |
|  | Percentage of commercial land | Australian Bureau of Statistics. Statistical Geography Volume 1 - Australian Standard Geographical Classification (ASGC).  Australian Bureau of Statistics. Australian Statistical Geography Standard (ASGS) Volume 1 - Main Structure and Greater Capital City Statistical Areas. | 2006 [W1-W2]  2011 [W3-W4]  2016 [W5-W7] |
| Natural environment | Percentage of parkland | Australian Bureau of Statistics. Statistical Geography Volume 1 - Australian Standard Geographical Classification (ASGC)  Australian Bureau of Statistics. Australian Statistical Geography Standard (ASGS) Volume 1 - Main Structure and Greater Capital City Statistical Areas. | 2006 [W1-W2]  2011 [W3-W4]  2016 [W5-W7] |
|  | Percentage of tree cover | PMSA Australia Limited. GeoScape – Trees. | 2018 [W1-W5]  2019 [W6-W7] |
|  | Percentage of blue space | Geoscience Australia. Blue space.  PSMA Australia Limited. Hydrology.  Australian Hydrological Geospatial Fabric, Australian State & Territory (for coast). | 2006 (invariant features)  [W1-W7] |
| Ambient air pollution | Annual average NO_2_ | National-scale satellite-based land-use regression models for Australia  Knibbs LD, Hewson MG, Bechle MJ, Marshall JD, Barnett AG. A national satellite based land-use regression model for air pollution exposure assessment in Australia. Environ Res. 2014;135:204-11. | 2005-2006 [W1-W2]  2007-2011 [W3-W4]  2016-2019 [W5-W7] |
|  | Annual average PM_2.5_ | National-scale satellite-based land-use regression models for Australia  Knibbs LD et al. A national satellite based land-use regression model for air pollution exposure assessment in Australia. Environ Res. 2014;135:204-11.  Knibbs LD et al. Satellite-Based Land-Use Regression for Continental-Scale Long-Term Ambient PM_2.5_ Exposure Assessment in Australia. Environ Sci Technol. 2018;52(21):12445-55. | 2005-2006 [W1-W2]  2007-2011 [W3-W4]  2016-2019 [W5-W7] |

**Figure S1.** Geographical distribution of study participants (at the level of Statistical Areas 1 to preserve confidentiality)


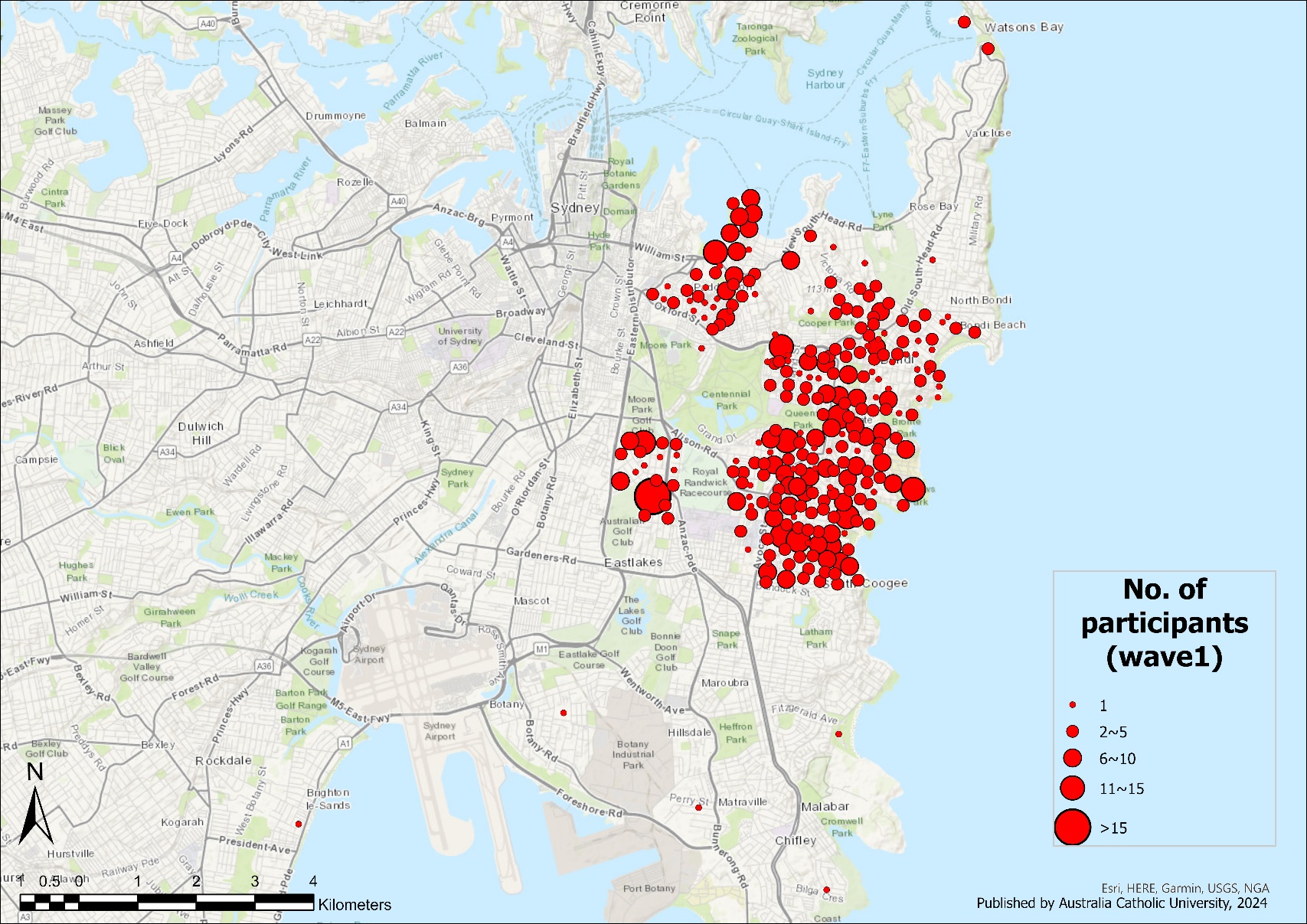


**Figure S2.** Directed acyclic graph (DAG) depicting the hypothesised relations between neighbourhood environmental attributes and cognitive state outcomes


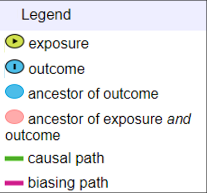

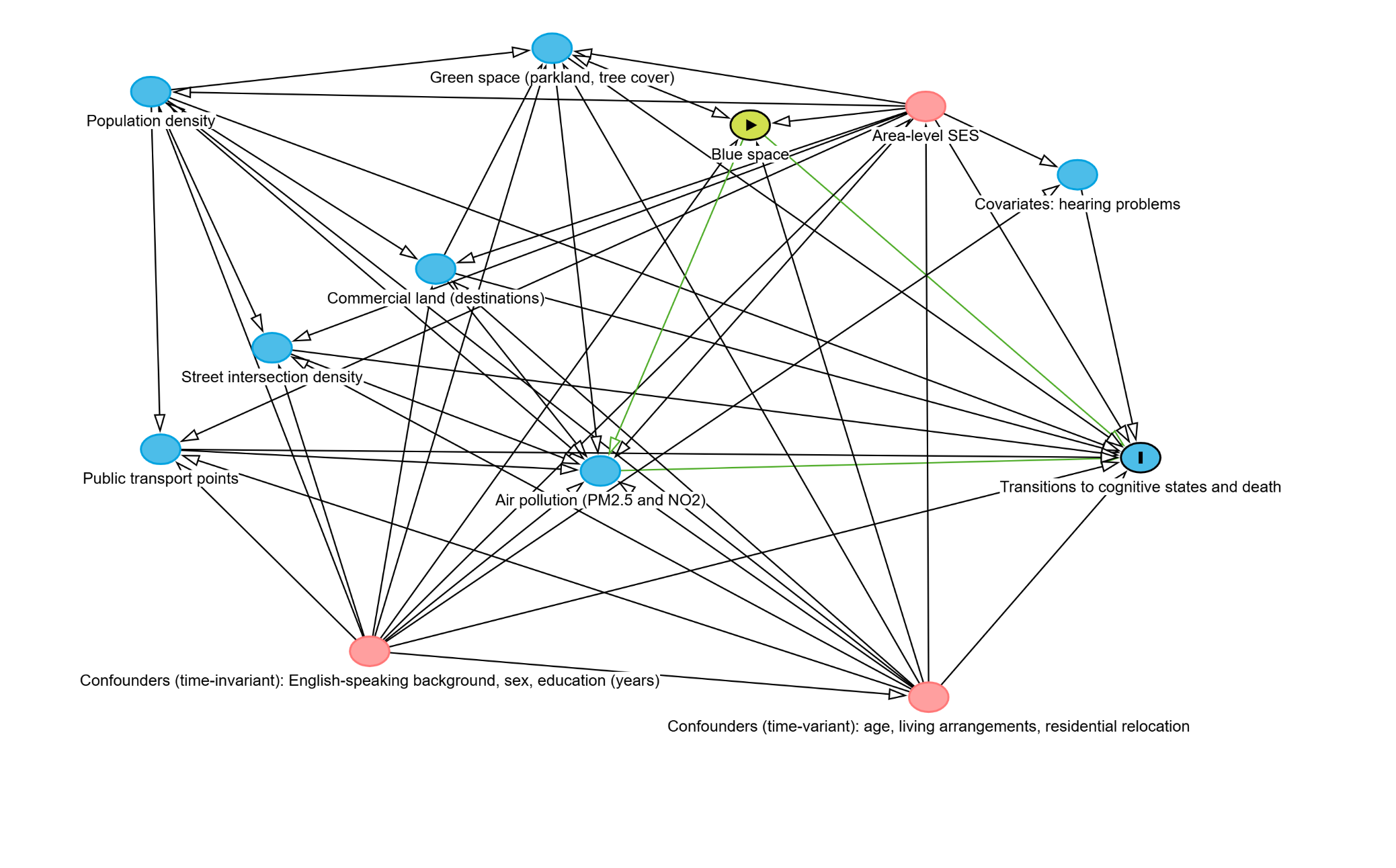


Directed acyclic graphs (DAGs) identified sets of variables to include in the statistical analyses to sufficiently control for potential confounders. The DAG above informed the model of the total effect of blue space in the residential buffer on transitions to cognitive states and death. Variables with red circles denote the set of potential confounders. SES = socio-economic status; PM_2.5_, particulate matter with a diameter of 2.5 μm or smaller; NO_2_, nitrogen dioxide.

**Table S2.** Confounders/covariates of multi-state models to estimate overall and independent effects of neighbourhood environmental attributes on transitions to cognitive states and death

| *Overall-effect models* |  | |
| --- | --- | --- |
| Attribute | Covariates | |
| Population density (persons/km^2^) | Area-level SES; Confounders (time-invariant): ESB, sex, education at baseline (years); Confounders (time-variant): age, living arrangements, residential relocation; Covariates: hearing problems | |
| Street intersection density (intersections/km^2^) | Area-level SES; Confounders (time-invariant): ESB, sex, education at baseline (years); Confounders (time-variant): age, living arrangements, residential relocation; Covariates: hearing problems; Population density | |
| Percentage of commercial land (% area in residential buffer) | As above | |
| Public transport points (number of stations/stops in the residential buffer) | As above | |
| Percentage of parkland (% of area in residential buffer) | Area-level SES; Confounders (time-invariant): ESB, sex, education at baseline (years); Confounders (time-variant): age, living arrangements, residential relocation; Covariates: hearing problems; Population density; Commercial land (destinations); Blue space | |
| Percentage of tree cover (% of area in residential buffer) | As above | |
| Percentage of blue space (% of area in residential buffer) | Area-level SES; Confounders (time-invariant): ESB, sex, education at baseline (years); Confounders (time-variant): age, living arrangements, residential relocation; Covariates: hearing problems; Population density; Commercial land (destinations); Parkland | |
| Annual average NO_2_ exposure (ppb) | Area-level SES; Confounders (time-invariant): ESB, sex, education at baseline (years); Confounders (time-variant): age, living arrangements, residential relocation; Covariates: hearing problems; Population density; Street intersection density; Commercial land (destinations); Parkland; Tree cover; Public transport points | |
| Annual average PM_2.5_ exposure (μg/m^3^) | As above | |
| *Independent-effect models* | |  |
| Population density (persons/km^2^) | Area-level SES; Confounders (time-invariant): ESB, sex, education at baseline (years); Confounders (time-variant): age, living arrangements, residential relocation; Covariates: hearing problems; Blue space; Commercial land (destinations); Street intersection density; Parkland; Tree cover; Public transport points; Air pollution (PM_2.5_ and NO_2_) | |
| Street intersection density (intersections/km^2^) | Area-level SES; Confounders (time-invariant): ESB, sex, education at baseline (years); Confounders (time-variant): age, living arrangements, residential relocation; Covariates: hearing problems; Commercial land (destinations); Population density; Parkland; Tree cover; Public transport points; Air pollution (PM_2.5_ and NO_2_) | |
| Percentage of commercial land (% area in residential buffer) | Area-level SES; Confounders (time-invariant): ESB, sex, education at baseline (years); Confounders (time-variant): age, living arrangements, residential relocation; Covariates: hearing problems; Blue space; Population density; Street intersection density; Parkland; Tree cover; Public transport points; Air pollution (PM_2.5_ and NO_2_) | |
| Public transport points (number of stations/stops in the residential buffer) | Area-level SES; Confounders (time-invariant): ESB, sex, education at baseline (years); Confounders (time-variant): age, living arrangements, residential relocation; Covariates: hearing problems; Population density; Street intersection density; Parkland; Tree cover; Commercial land (destination); Air pollution (PM_2.5_ and NO_2_) | |
| Percentage of parkland (% of area in residential buffer) | Area-level SES; Confounders (time-invariant): ESB, sex, education at baseline (years); Confounders (time-variant): age, living arrangements, residential relocation; Covariates: hearing problems; Population density; Street intersection density; Blue space; Tree cover; Commercial land (destination); Public transport points; Air pollution (PM_2.5_ and NO_2_) | |
| Percentage of tree cover (% of area in residential buffer) | Area-level SES; Confounders (time-invariant): ESB, sex, education at baseline (years); Confounders (time-variant): age, living arrangements, residential relocation; Covariates: hearing problems; Population density; Street intersection density; Blue space; Parkland; Commercial land (destination); Public transport points; Air pollution (PM_2.5_ and NO_2_) | |
| Percentage of blue space (% of area in residential buffer) | Area-level SES; Confounders (time-invariant): ESB, sex, education at baseline (years); Confounders (time-variant): age, living arrangements, residential relocation; Covariates: hearing problems; Population density; Street intersection density; Parkland; Tree cover; Commercial land (destination); Public transport points; Air pollution (PM_2.5_ and NO_2_) | |
| Annual average NO_2_ exposure (ppb) | Area-level SES; Confounders (time-invariant): ESB, sex, education at baseline (years); Confounders (time-variant): age, living arrangements, residential relocation; Covariates: hearing problems; Population density; Street intersection density; Blue space; Parkland; Tree cover; Commercial land (destination); Public transport points; Air pollution (PM_2.5_) | |
| Annual average PM_2.5_ exposure (μg/m^3^) | Area-level SES; Confounders (time-invariant): ESB, sex, education at baseline (years); Confounders (time-variant): age, living arrangements, residential relocation; Covariates: hearing problems; Population density; Street intersection density; Blue space; Parkland; Tree cover; Commercial land (destination); Public transport points; Air pollution (NO_2_) | |

*Notes.* ESB = English-speaking background; SES = socio-economic status; PM_2.5_, particulate matter with a diameter of 2.5 μm or smaller; NO_2_, nitrogen dioxide; ppb, parts per billion.

**Predictors of participants’ retention vs. drop-out (withdrawal or loss to follow-up) from the study and calculation of inverse probability weights**

Backward stepwise logistic regression models were used to determine baseline predictors of remaining vs. not remaining in the study at a specific study wave due to reasons other than death (withdrawing from the study or being lost to follow-up). As death was considered a transition state (outcome), it was not treated as reason for attrition. Predictors included in the models were participants’ time-invariant socio-demographic characteristics (sex, English-speaking background, years of education and weekly hours of work at baseline), and time-variant socio-demographic and health-related characteristics at the previous assessment/wave [age, living arrangements, using a hearing aid, being mobile without the assistance of mobility aids, activities of daily living (ADLs; as measured by Bayer-ADL [14]), instrumental activities of daily living (IADLs [15]), depressive symptoms as measured by the 15-item Geriatric Depression Scale (GDS [16]) and cognitive state (no dementia vs dementia for Waves 6 to 7; normal cognition vs. mild cognitive impairment vs. dementia for Waves 2 to 5)]. We considered non-linear relationships and theoretically plausible interactions between variables.

**Table S3.** Predictors of participants’ remaining in the study at Waves 2 to 7

| **Predictors** | **OR** | **95% CI** | ***p*-value** |
| --- | --- | --- | --- |
| *Predictors of retention at Wave 2* |  |  |  |
| Education (years) | 1.10 | (1.02, 1.19) | 0.013 |
| Using a hearing aid | 4.12 | (2.03, 8.36) | <0.001 |
| Mobile without mobility aids | 8.03 | (4.45, 14.51) | <0.001 |
| Cognitive state (ref: normal cognition) |  |  |  |
| Mild cognitive impairment | 0.60 | (0.35, 1.03) | 0.062 |
| Unclassifiable | 0.48 | (0.26, 0.91) | 0.025 |
| *Predictors of retention at Wave 3* |  |  |  |
| Mobile without mobility aids | 1.87 | (1.03, 3.38) | 0.039 |
| Cognitive state (ref: normal cognition) |  |  |  |
| Mild cognitive impairment | 0.56 | (0.28, 1.15) | 0.116 |
| Dementia | 0.23 | (0.06, 0.87) | 0.031 |
| Unclassifiable | 0.35 | (0.17, 0.72) | 0.004 |
| *Predictors of retention at Wave 4* |  |  |  |
| Education (years) | 1.13 | (1.01, 1.27) | 0.037 |
| Cognitive state (ref: normal cognition) |  |  |  |
| Mild cognitive impairment | 2.47 | (0.72, 8.54) | 0.152 |
| Dementia | 1.81 | (0.23, 13.96) | 0.571 |
| Unclassifiable | 0.44 | (0.21, 0.91) | 0.027 |
| *Predictors of retention at Wave 5* |  |  |  |
| Education (years) | 1.12 | (1.01, 1.25) | 0.038 |
| Cognitive state (ref: normal cognition) |  |  |  |
| Mild cognitive impairment | 0.59 | (0.28, 1.25) | 0.170 |
| Dementia | 0.40 | (0.15, 1.11) | 0.078 |
| Unclassifiable | 0.17 | (0.05, 0.59) | 0.005 |
| *Predictors of retention at Wave 6* |  |  |  |
| Cognitive state (ref: no dementia) |  |  |  |
| Dementia | 0.63 | (0.21, 1.92) | 0.419 |
| Unclassifiable | 0.08 | (0.01, 0.53) | 0.008 |
| *Predictors of retention at Wave 7* |  |  |  |
| Cognitive state (ref: no dementia) |  |  |  |
| Dementia | 0.78 | (0.32, 1.90) | 0.581 |
| Unclassifiable | 0.09 | (0.01, 0.70) | 0.021 |

*Notes.* Results of the final model including only statistically significant predictors. OR = odds ratio; CI = confidence intervals; ref: reference category

To obtain inverse probability weights for the multi-state models estimated in this study, we calculated the conditional probability (propensity score) of remaining in the study at a specific wave based on the final logistic regression models shown in Table S3 and then calculated the inverse of the propensity score.

**Associations among neighbourhood environmental attributes**

Associations between neighbourhood environmental attributes were estimated using generalised linear mixed models (GAMMs; package ‘mgcv’ version 1.8.42 in R) with Gaussian variance and identity link functions, and random intercept at the person level, accounting for dependency in the data at the participant level (repeated measures of environmental characteristics) [17]. Curvilinear relationships were modelled using semi-parametric smooth terms estimated with thin plate splines. The choice of a linear vs curvilinear (smooth) term for specific covariates was based on Akaike Information Criterion (AIC) values [17]. A ≥ 5-unit difference in AIC between GAMMs with a linear vs. curvilinear term was as the criterion for model selection, with lower AIC values indicating better fitting and, hence, preferred models [18].

**Table S4.** Confounders included in generalised additive mixed models to estimate associations (independent effects) among neighbourhood environmental attributes based on the directed acyclic graph depicted in Figure S2

| Attribute | Covariates |
| --- | --- |
| Street intersection density (intersections/km^2^) | Area-level SES; Confounders (time-invariant): ESB, sex, education at baseline (years); Confounders (time-variant): age, living arrangements, residential relocation; Population density |
| Percentage of commercial land (% area in residential buffer) | As above |
| Public transport points (number of stations/stops in the residential buffer) | As above |
| Percentage of parkland (% of area in residential buffer) | Area-level SES; Confounders (time-invariant): ESB, sex, education at baseline (years); Confounders (time-variant): age, living arrangements, residential relocation; Population density; Commercial land (destinations) |
| Percentage of tree cover (% of area in residential buffer) | As above |
| Percentage of blue space (% of area in residential buffer) | Area-level SES; Confounders (time-invariant): ESB, sex, education at baseline (years); Confounders (time-variant): age, living arrangements, residential relocation; Population density |
| Annual average NO_2_ exposure (ppb) | Area-level SES; Confounders (time-invariant): ESB, sex, education at baseline (years); Confounders (time-variant): age, living arrangements, residential relocation; Population density; Street intersection density; Commercial land (destinations); Parkland; Tree cover; Public transport points; Blue space |
| Annual average PM_2.5_ exposure (μg/m^3^) | As above |

*Notes.* ESB = English-speaking background; SES = socio-economic status; PM_2.5_, particulate matter with a diameter of 2.5 μm or smaller; NO_2_, nitrogen dioxide; ppb, parts per billion.

**Figure S3.** Associations between neighbourhood environmental attributes (original estimates of exposure)


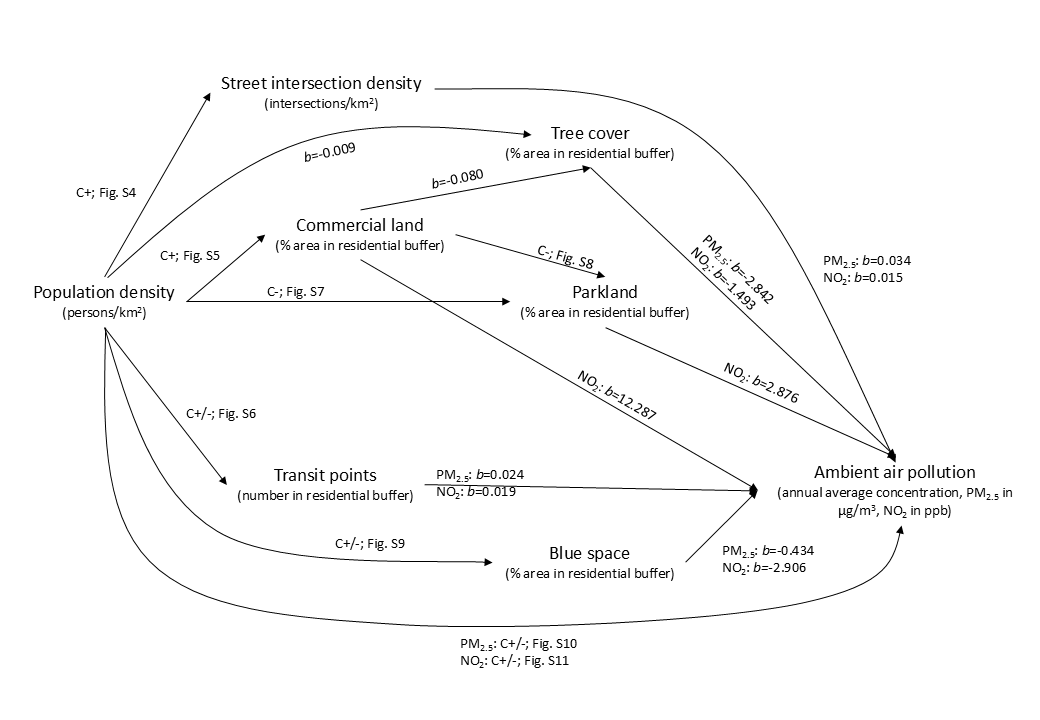


*Notes.* Arrows linking variables indicate associations, all of which were significant at the 0.001 probability level. *b*, regression coefficient; PM_2.5_, particulate matter with a diameter of 2.5 μm or smaller; NO_2_, nitrogen dioxide; ppb, parts per billion; C+, curvilinear positive relationship; C+/-, curvilinear non-monotonic associations. The curvilinear associations are depicted in Figures S4 to S11. The distribution of the data for the environmental covariates is visible from the rug plots of the x-axis of the graphs (represented by tick marks or small lines, which denote individual data points). The correlation between air pollutants was 0.492.

**Figure S4.** Curvilinear relationship of population density with street intersection density.


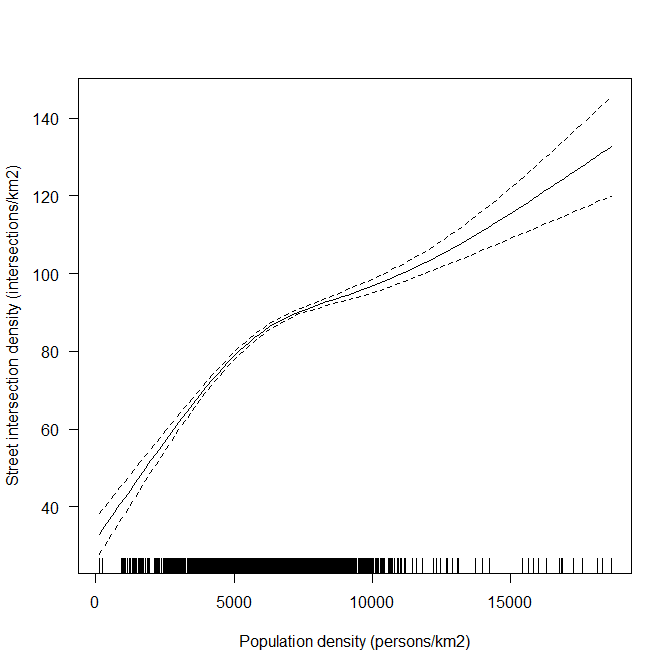


**Figure S5.** Curvilinear relationship of population density with percentage of commercial land


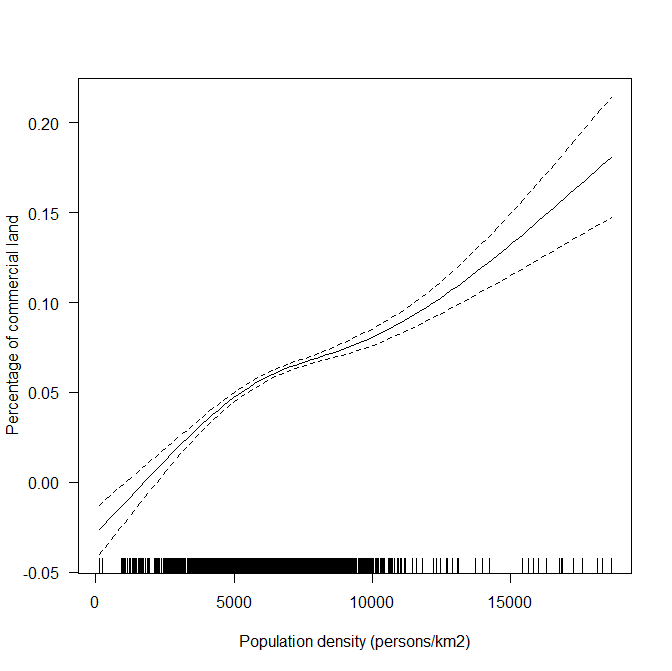


**Figure S6.** Curvilinear relationship of population density with number of transit points


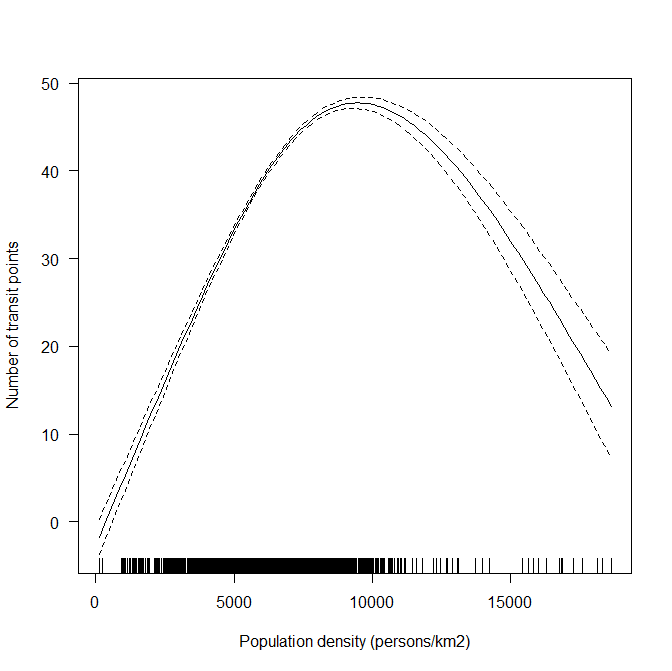


**Figure S7.** Curvilinear relationship of population density with percentage of parkland


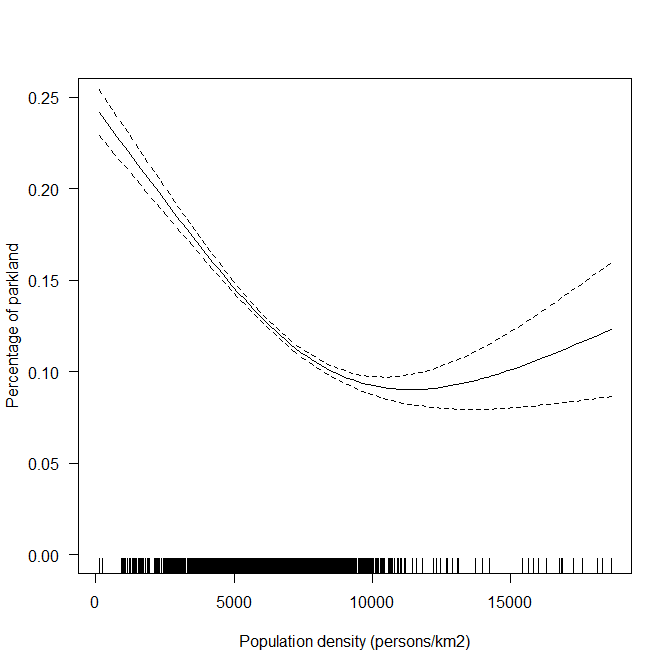


**Figure S8.** Curvilinear relationship of percentage of commercial land with percentage of parkland


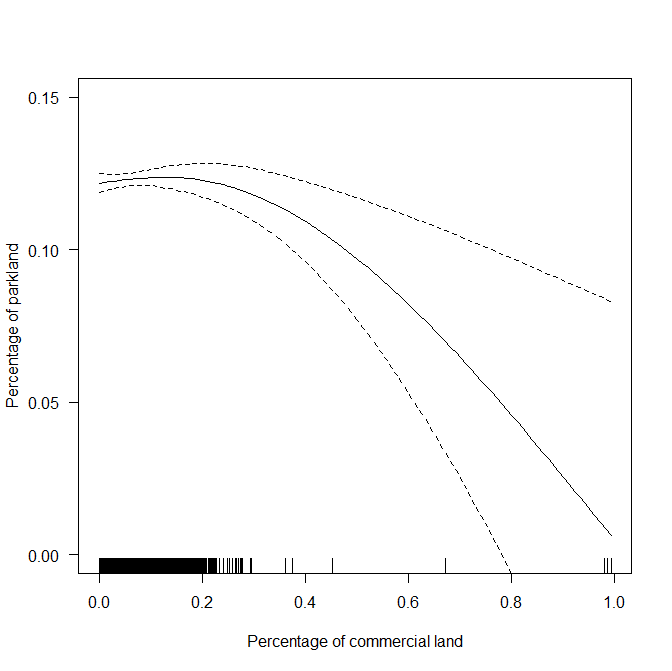


**Figure S9.** Curvilinear relationship of population density with percentage of blue space


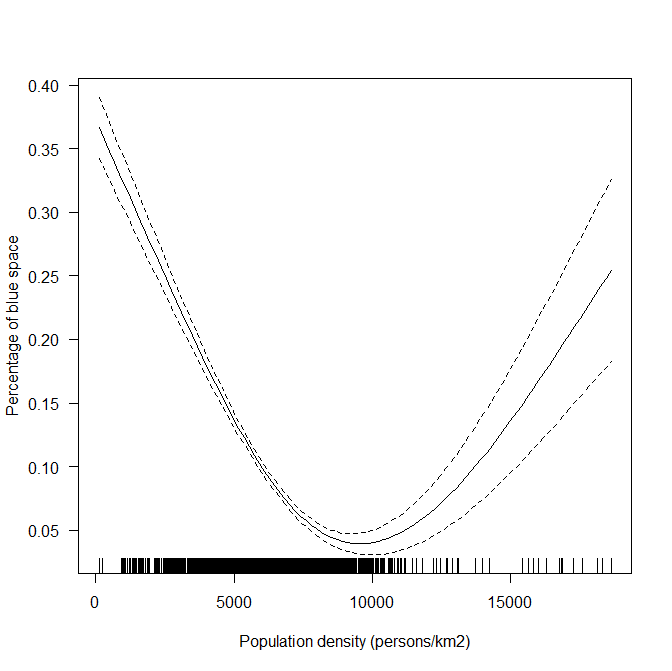


**Figure S10.** Curvilinear relationship of population density with annual average concentrations of PM_2.5_


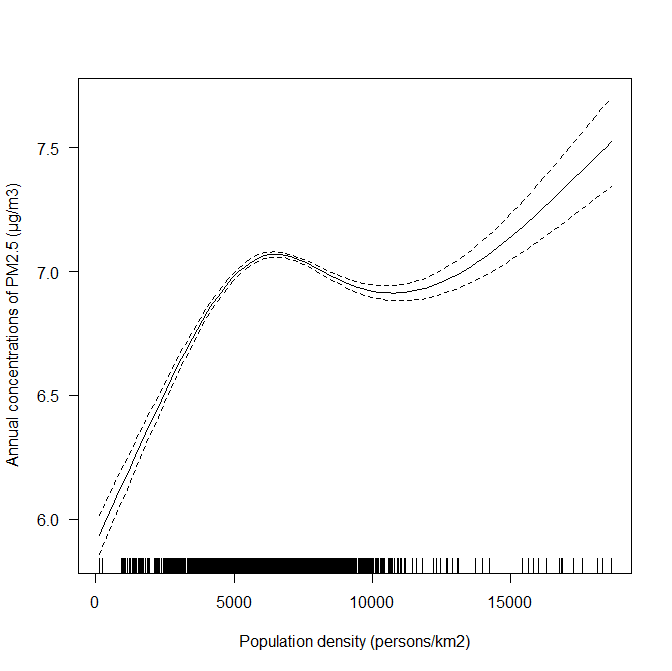


**Figure S11.** Curvilinear relationship of population density with annual average concentrations of NO_2_


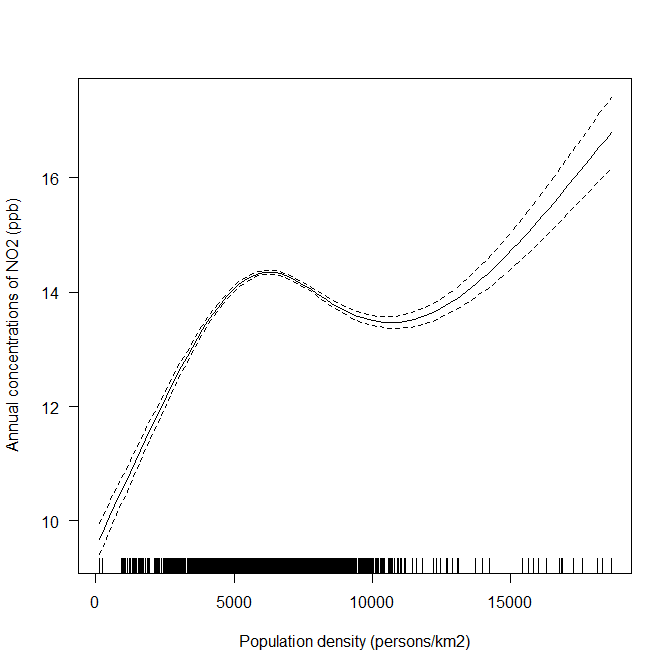


**Figure S12.** Associations between neighbourhood environmental attributes (estimates of cumulative exposure)


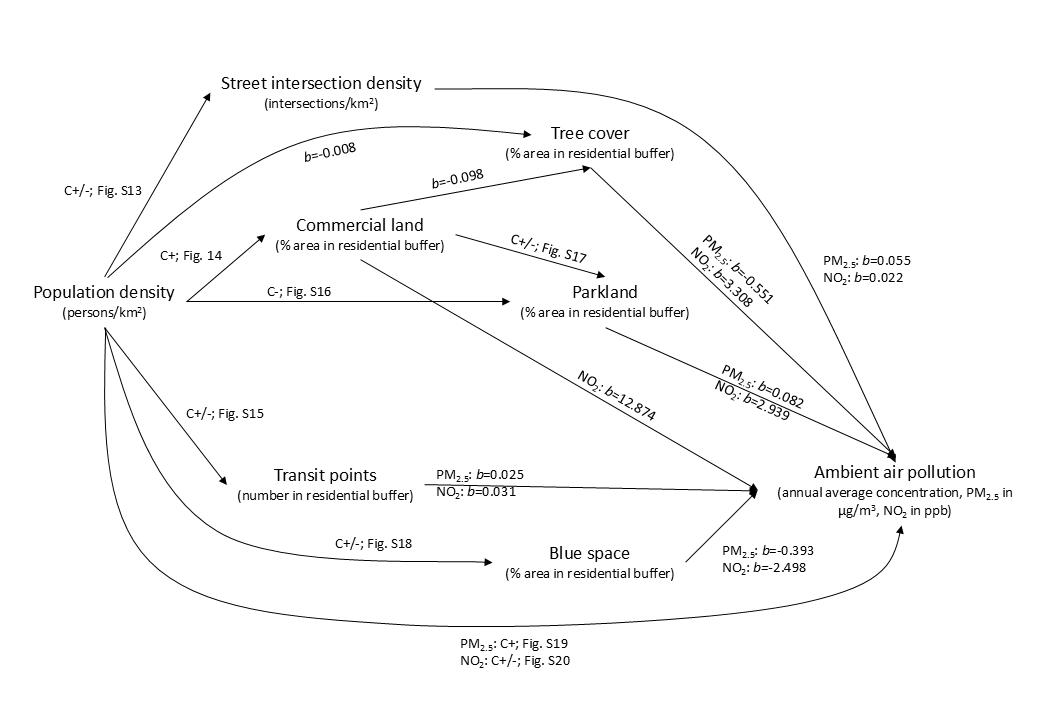


*Notes.* Arrows linking variables indicate associations, all of which were significant at the 0.001 probability level. *b*, regression coefficient; PM_2.5_, particulate matter with a diameter of 2.5 μm or smaller; NO_2_, nitrogen dioxide; ppb, parts per billion; C+, curvilinear positive relationship; C+/-, curvilinear non-monotonic associations. The curvilinear associations are depicted in Figures S13 to S20. The distribution of the data for the environmental covariates is visible from the rug plots of the x-axis of the graphs (represented by tick marks or small lines, which denote individual data points). The correlation between air pollutants was 0.601.

**Figure S13.** Curvilinear relationship of population density with street intersection density


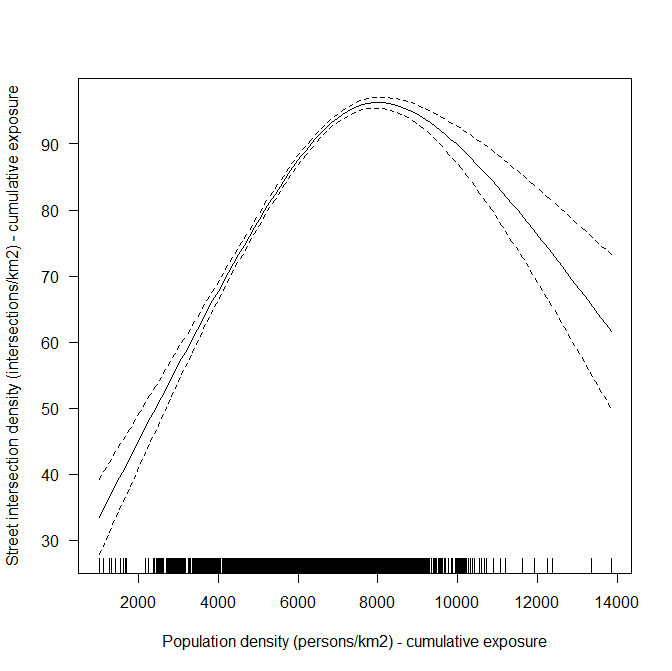


**Figure S14.** Curvilinear relationship of population density with percentage of commercial land


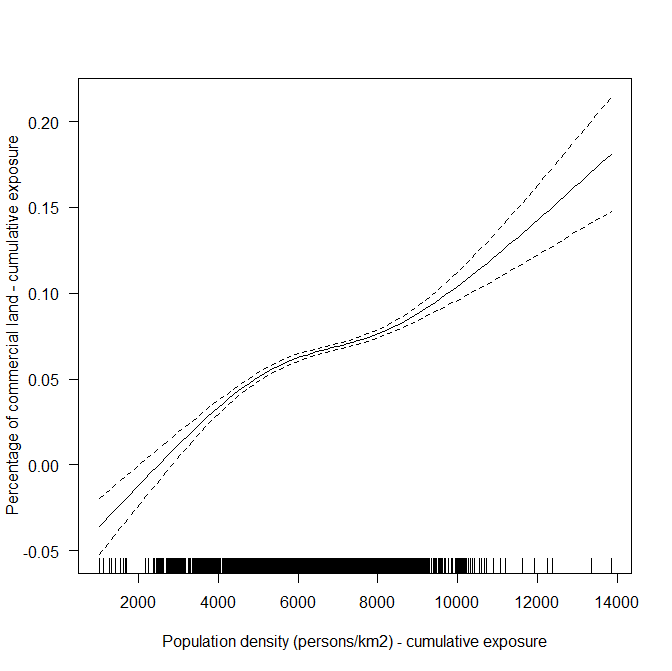


**Figure S15.** Curvilinear relationship of population density with number of transit points


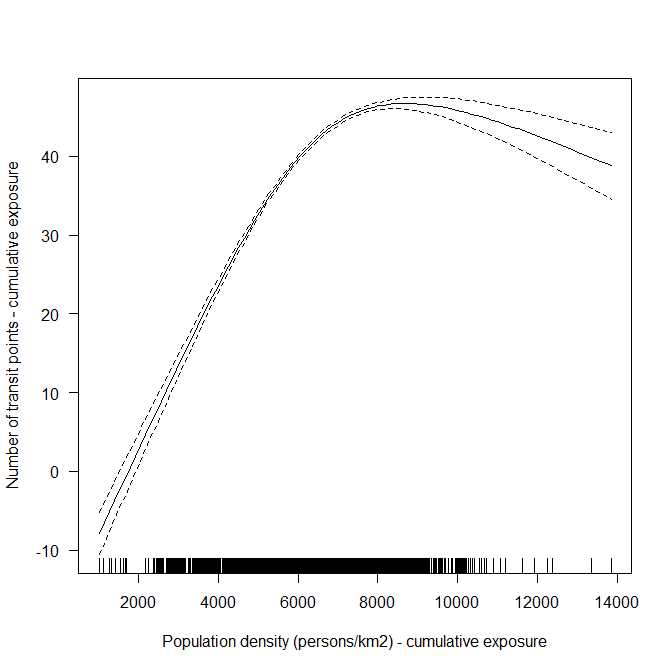


**Figure S16.** Curvilinear relationship of population density with percentage of parkland


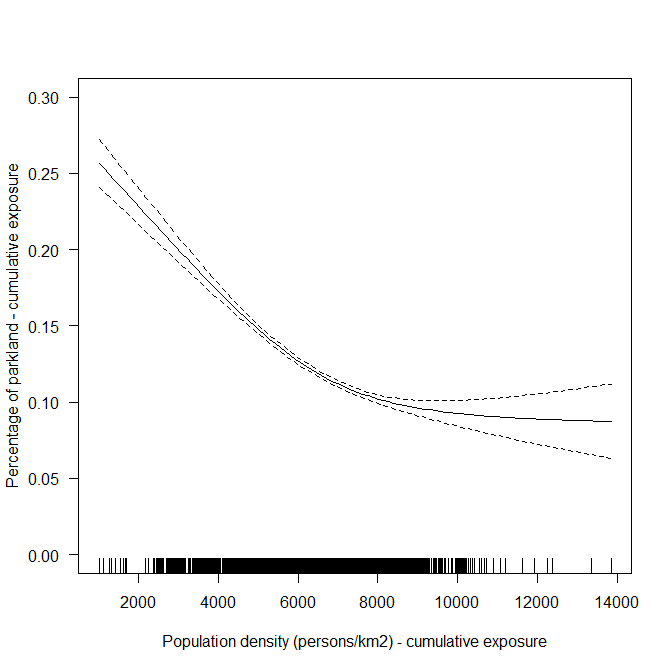


**Figure S17.** Curvilinear relationship of percentage of commercial land with percentage of parkland


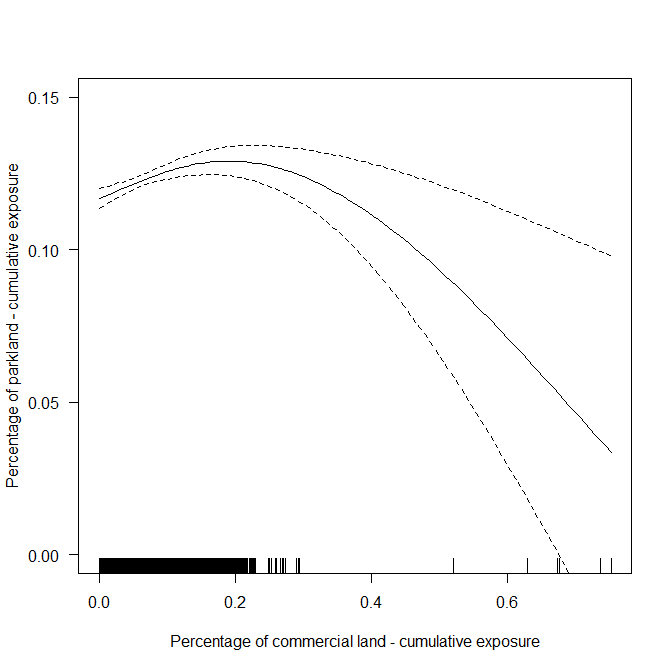


**Figure S18.** Curvilinear relationship of population density with percentage of blue space


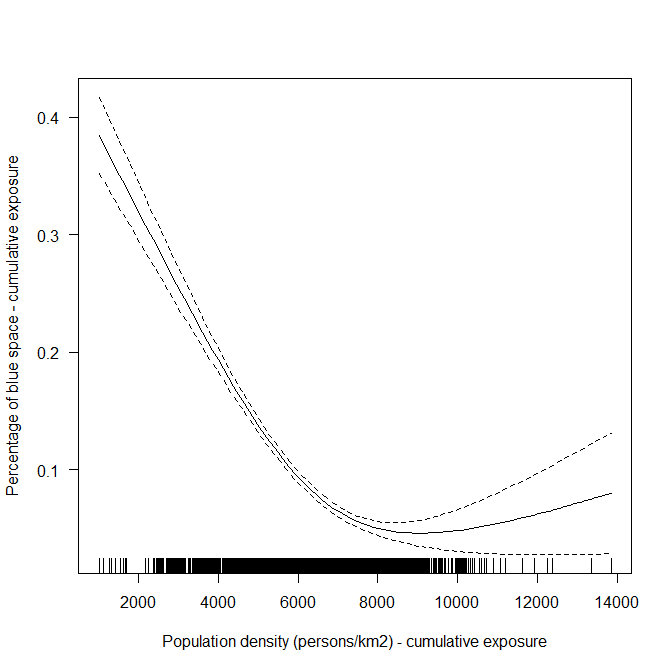


**Figure S19.** Curvilinear relationship of population density with annual average concentrations of PM_2.5_


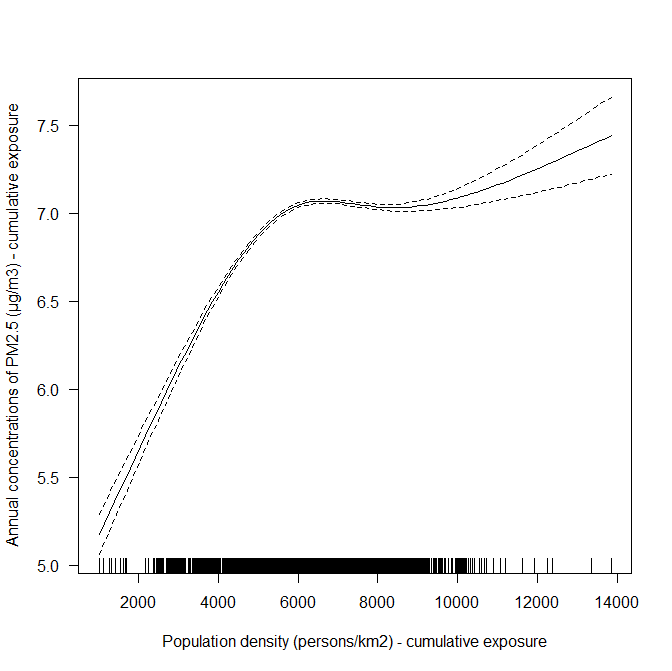


**Figure S20.** Curvilinear relationship of population density with annual average concentrations of NO_2_


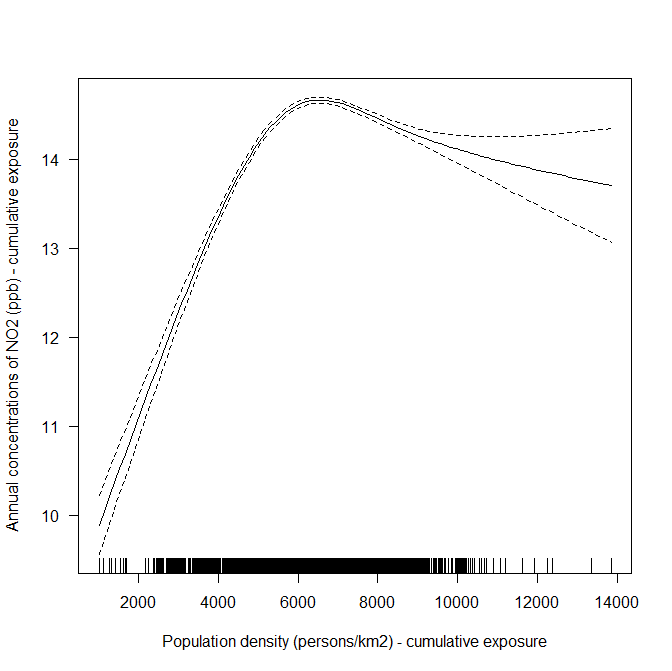


**Table S5.** Neighbourhood environmental attributes as correlates of transitions from no dementia and dementia to death: overall- and independent-effect models (N = 1036 followed up for up to 13.7 years)

|  | Spatial indicator | Overall-effect models | Independent-effect models | Overall-effect models | Independent-effect models |
| --- | --- | --- | --- | --- | --- |
| Neighbourhood environmental attribute |  | No dementia to death | No dementia to death | Dementia to death | Dementia to death |
|  |  | HR (95% CI) | HR (95% CI) | HR (95% CI) | HR (95% CI) |
| Population density (100 persons/km^2^) | Original | 1.003 (0.992, 1.009) | 0.998 (0.997, 1.002) | **0.992 (0.984, 0.999)** | **0.989 (0.979, 0.999)** |
|  | Cumulative | 1.000 (0.987, 1.013) | 0.997 (0.985, 1.009) | 1.001 (0.090, 1.013) | 0.997 (0.983, 1.012) |
| Street intersection density (intersections/km^2^) | Original | **1.008 (1.000, 1.015)** | **1.012 (1.005, 1.018)** | 1.004 (0.996, 1.011) | 0.999 (0.992, 1.007) |
|  | Cumulative | **1.009 (1.000, 1.017)** | 1.005 (0.999, 1.010) | 1.004 (0.994, 1.014) | 1.004 (0.997, 1.010) |
| Transit points (count within buffer) | Original | 1.002 (0.987, 1.017) | 1.004 (0.986, 1.021) | 1.004 (0.989, 1.019) | 1.000 (0.984, 1.017) |
|  | Cumulative | 1.003 (0.988, 1.019) | 1.002 (0.985, 1.020) | 1.003 (0.988, 1.017) | 1.009 (0.991, 1.028) |
| Commercial land (% within buffer) | Original | 1.015 (0.995, 1.034) | 1.004 (0.979, 1.030) | 1.001 (0.973, 1.030) | 0.992 (0.960, 1.024) |
|  | Cumulative | 1.021 (0.999, 1.043) | 1.017 (0.988, 1.046) | 0.994 (0.959, 1.030) | 0.964 (0.918, 1.013) |
| Parkland (% within buffer) | Original | 1.001 (0.974, 1.030) | 0.995 (0.966, 1.024) | 0.995 (0.976, 1.015) | 0.998 (0.975, 1.021) |
|  | Cumulative | 1.000 (0.974, 1.026) | 1.003 (0.975, 1.032) | 0.999 (0.976, 1.026) | 0.989 (0.963, 1.017) |
| Tree cover (% within buffer) | Original | 0.988 (0.948, 1.030) | 0.999 (0.952, 1.049) | 0.984 (0.957, 1.012) | 0.982 (0.953, 1.011) |
|  | Cumulative | 0.986 (0.942, 1.033) | 0.983 (0.930, 1.038) | 0.974 (0.936, 1.014) | 0.973 (0.928, 1.020) |
| Blue space (% within buffer) | Original | 1.000 (0.987, 1.012) | 1.007 (0.990, 1.024) | 0.998 (0.985, 1.011) | 0.998 (0.983, 1.012) |
|  | Cumulative | 0.998 (0.984, 1.012) | 1.001 (0.982, 1.021) | 1.006 (0.993, 1.020) | 1.012 (0.995, 1.029) |
| NO_2_ (ppb) | Original | 1.130 (0.971, 1.316) | 1.159 (0.984, 1.365) | 1.005 (0.897, 1.124) | 1.003 (0.880, 1.143) |
|  | Cumulative | 1.040 (0.876, 1.232) | 1.080 (0.881, 1.324) | 1.045 (0.893, 1.223) | 1.155 (0.938, 1.422) |
| PM_2.5_ (µg/m^3^) | Original | 1.047 (0.591, 1.857) | 0.946 (0.496, 1.804) | 1.008 (0.722, 1.406) | 0.980 (0.669, 1.434) |
|  | Cumulative | 0.840 (0.450, 1.533) | 0.761 (0.391, 1.481) | 0.922 (0.593, 1.435) | 0.795 (0.456, 1.385) |

*Notes.* HR, hazard ratio; CI, confidence interval; PM_2.5_, particulate matter with a diameter of 2.5 μm (micrometres) or smaller; NO_2_, nitrogen dioxide; ppb, parts per billion. Estimates adjusted for confounders. Inverse probability weights were used to account for attrition bias. Statistically significant effects are in bold. 81 out of 4154 observations (1.9%) had insufficient cognitive state data. 279 transitions from no dementia to dementia were recorded; 126 transitions from dementia to death were recorded.

**Table S6.** Neighbourhood environmental attributes as correlates of transitions from normal cognition, mild cognitive impairment (MCI) or dementia to death: overall- and independent-effect models (N = 1036 followed up for ~6 years)

|  |  | Overall-effect models | Independent-effect models | Overall-effect models | Independent-effect models | Overall-effect models | Independent-effect models |
| --- | --- | --- | --- | --- | --- | --- | --- |
| Neighbourhood environmental attribute | Spatial indicator | Normal cognition to death | Normal cognition to death | MCI to death | MCI to death | Dementia to death | Dementia to death |
|  |  | HR (95% CI) | HR (95% CI) | HR (95% CI) | HR (95% CI) | HR (95% CI) | HR (95% CI) |
| Population density (100 persons/km^2^) | Original | 0.992 (0.967, 1.019) | 1.001 (0.966, 1.038) | 0.994 (0.967, 1.021) | 1.003 (0.946, 1.062) | 1.000 (0.976, 1.023) | 0.997 (0.967, 1.025) |
|  | Cumulative | 0.992 (0.970, 1.014) | 0.997 (0.957, 1.038) | 0.999 (0.965, 1.035) | 0.993 (0.931, 1.057) | 1.002 (0.970, 1.036) | 1.015 (0.974, 1.058) |
| Street intersection density (intersections/km^2^) | Original | 0.997 (0.980, 1.015) | 1.004 (0.981, 1.028) | 1.008 (0.986, 1.031) | 0.943 (0.875, 1.015) | **1.015 (1.002, 1.029)** | 1.015 (0.993, 1.036) |
|  | Cumulative | 0.999 (0.979, 1.019) | 1.018 (0.993, 1.043) | 1.012 (0.990, 1.034) | 0.920 (0.842, 1.005) | 1.004 (0.988, 1.020) | 1.009 (0.986, 1.032) |
| Transit points (count) | Original | 0.989 (0.965, 1.013) | 0.982 (0.945, 1.020) | 0.919 (0.791, 1.067) | **1.081 (1.008, 1.160)** | 1.009 (0.984, 1.034) | 1.015 (0.981, 1.051) |
|  | Cumulative | 0.989 (0.965, 1.013) | 0.985 (0.950, 1.021) | 0.937 (0.793, 1.107) | 1.043 (0.976, 1.114) | 1.004 (0.977, 1.031) | 1.024 (0.988, 1.062) |
| Commercial land (%) | Original | 1.025 (0.992, 1.060) | 1.023 (0.985, 1.063) | **0.880 (0.775, 0.999)** | 1.250 (0.949, 1.063) | **1.034 (1.011, 1.057)** | 0.964 (0.879, 1.056) |
|  | Cumulative | 1.020 (0.968, 1.074) | 1.044 (0.992, 1.097) | 0.896 (0.776, 1.033) | 1.191 (0.937, 1.514) | **1.042 (1.020, 1.065)** | 0.956 (0.862, 1.060) |
| Parkland (%) | Original | 1.009 (0.963, 1.057) | 1.016 (0.970, 1.065) | 0.994 (0.925, 1.068) | 0.992 (0.937, 1.051) | 1.027 (0.992, 1.064) | 1.002 (0.962, 1.043) |
|  | Cumulative | 0.970 (0.913, 1.030) | 1.007 (0.952, 1.065) | 1.013 (0.947, 1.084) | 0.992 (0.935, 1.053) | 1.018 (0.981, 1.057) | 1.013 (0.965, 1.063) |
| Tree cover (%) | Original | 0.969 (0.891, 1.054) | 0.975 (0.879, 1.082) | 1.099 (0.984, 1.227) | 1.296 (0.950, 1.767) | 0.936 (0.853, 1.027) | 0.951 (0.849, 1.078) |
|  | Cumulative | 0.969 (0.882, 1.065) | 0.960 (0.852, 1.081) | 1.067 (0.908, 1.254) | 1.184 (0.932, 1.503) | 0.990 (0.884, 1.108) | 1.017 (0.889, 1.164) |
| Blue space (%) | Original | 1.009 (0.990, 1.027) | 1.008 (0.978, 1.039) | 1.004 (0.967, 1.041) | 1.038 (0.967, 1.041) | 0.994 (0.969, 1.020) | 1.007 (0.972, 1.042) |
|  | Cumulative | 1.015 (0.997, 1.034) | 1.006 (0.975, 1.037) | 0.981 (0.893, 1.078) | 1.028 (0.972, 1.015) | 1.007 (0.986, 1.028) | 1.018 (0.985, 1.016) |
| NO_2_ (ppb) | Original | 0.990 (0.733, 1.339) | 1.213 (0.800, 1.839) | 0.908 (0.567, 1.454) | 0.344 (0.074, 1.591) | 1.179 (0.929, 1.497) | 1.291 (0.862, 1.932) |
|  | Cumulative | 0.926 (0.665, 1.290) | 1.037 (0.688, 1.564) | 0.500 (0.135,1.851) | 0.432 (0.091, 2.055) | 1.171 (0.832, 1.648) | 1.359 (0.806, 2.293) |
| PM_2.5_ (µg/m^3^) | Original | 2.585 (0.664, 10.056) | 2.385 (0.747, 7.616) | 1.512 (0.171, 13.376) | 3.132 (0.941, 10.422) | 1.120 (0.432, 2.904) | 1.290 (0.267, 6.226) |
|  | Cumulative | 2.743 (0.616, 12.206) | 2.422 (0.913, 6.428) | 1.957 (0.311, 12.304) | 2.735 (0.941, 7.950) | 2.116 (0.714, 6.266) | 1.433 (0.329, 6.245) |

*Notes.* HR, hazard ratio; CI, confidence interval; MCI, mild cognitive impairment; PM_2.5_, particulate matter with a diameter of 2.5 μm (micrometres) or smaller; NO_2_, nitrogen dioxide; ppb, parts per billion. Estimates adjusted for confounders. Inverse probability weights were used to account for attrition bias. Statistically significant effects are in bold. 474 out of 2530 observations (18.7%) with insufficient cognitive state data. 64, 49 and 19 transitions from normal cognition, MCI and dementia to death were, respectively, recorded.

**Sensitivity analyses – scenarios for unclassifiable cognitive states**

We applied two scenarios to replace unclassifiable cognitive states with the following:

**Scenario 1** (optimistic): We assumed that the unclassifiable cognitive state was ‘no dementia’ for those individuals who had a ‘no dementia’ diagnosis in the previous wave (for the simpler classification of cognition states using the categories ‘no dementia’ and ‘dementia’); and ‘normal cognition’ in those who had a previous diagnosis of ‘normal cognition’ or ‘mild cognitive impairment’ in the previous wave (for the more fine-grained classification of cognitions states using the categories ‘normal state’, ‘MCI’ and ‘dementia’. Results are presented in Tables S7-S11.

**Scenario 2** (pessimistic): We assumed that unclassifiable cognitive states were due to a decline in cognition (i.e., dementia or MCI, as appropriate). Results are presented in Tables S7-S11.

**Table S7.** Neighbourhood environmental attributes as correlates of transitions from no dementia to dementia: overall- and independent-effect models for Scenario 1 and 2 (N = 1036 followed up for up to 13.7 years)

|  |  | Scenario 1 (optimistic) | | Scenario 2 (pessimistic) | |
| --- | --- | --- | --- | --- | --- |
| Neighbourhood environmental attribute |  | Overall-effect models | Independent-effect models | Overall-effect models | Independent-effect models |
|  | Spatial indicator | HR (95% CI) | HR (95% CI) | HR (95% CI) | HR (95% CI) |
| Population density (100 persons/km^2^) | Original | 1.008 (0.993, 1.022) | 0.996 (0.987, 1.004) | 0.996 (0.985, 1.006) | 0.995 (0.989, 1.002) |
|  | Cumulative | 1.001 (0.993, 1.009) | 0.997 (0.984, 1.011) | 0.998 (0.987, 1.010) | 1.000 (0.987, 1.013) |
| Street intersection density (intersections/km^2^) | Original | 1.001 (0.990, 1.011) | 1.001 (0.989, 1.013) | 0.998 (0.988, 1.008) | 1.004 (0.996, 1.011) |
|  | Cumulative | 0.999 (0.994, 1.004) | 0.997 (0.989, 1.005) | 1.001 (0.996, 1.005) | 1.004 (0.999, 1.010) |
| Transit points (count) | Original | **0.992 (0.984, 0.999)** | **0.989 (0.978, 0.999)** | 0.994 (0.987, 1.003) | 0.992 (0.981, 1.003) |
|  | Cumulative | 0.993 (0.983, 1.004) | **0.990 (0.980, 0.999)** | 0.998 (0.982, 1.011) | 0.993 (0.983, 1.004) |
| Commercial land (%) | Original | **0.944 (0.893, 0.998)** | 0.996 (0.971, 1.021) | **0.991 (0.984, 0.999)** | 0.998 (0.991, 1.006) |
|  | Cumulative | **0.978 (0.957, 0.999)** | 0.975 (0.943, 1.006) | 0.995 (0.992, 1.000) | 0.992 (0.975, 1.009) |
| Parkland (%) | Original | 0.994 (0.988, 1.002) | 1.003 (0.979, 1.026) | **0.971 (0.944, 0.999)** | 0.993 (0.975, 1.011) |
|  | Cumulative | 0.996 (0.978, 1.016) | 0.997 (0.979, 1.015) | 0.973 (0.945, 1.003) | 0.981 (0.974, 1.006) |
| Tree cover (%) | Original | **0.960 (0.934, 0.986)** | **0.955 (0.929, 0.983)** | **0.948 (0.910, 0.988)** | **0.954 (0.920, 0.987)** |
|  | Cumulative | **0.976 (0.952, 0.999)** | **0.973 (0.950, 0.997)** | **0.971 (0.942, 0.998)** | **0.968 (0.938, 0.999)** |
| Blue space (%) | Original | 1.001 (0.991, 1.010) | 0.995 (0.982, 1.009) | 1.002 (0.992, 1.011) | 0.984 (0.961, 1.008) |
|  | Cumulative | 1.009 (0.997, 1.022) | 0.998 (0.976, 1.021) | 1.011 (0.999, 1.023) | 0.999 (0.974, 1.025) |
| NO_2_ (ppb) | Original | **0.901 (0.821, 0.999)** | **0.887 (0.809, 0.973)** | 0.994 (0.893, 1.107) | 0.966 (0.858, 1.087) |
|  | Cumulative | 1.015 (0.903, 1.139) | 0.971 (0.841, 1.121) | 1.073 (0.924, 1.245) | 1.005 (0.897, 1.124) |
| PM_2.5_ (µg/m^3^) | Original | 1.017 (0.746, 1.387) | 1.183 (0.628, 2.226) | 1.008 (0.871, 1.166) | 1.073 (0.924, 1.245) |
|  | Cumulative | **1.575 (1.313, 1.890)** | **1.640 (1.402, 1.918)** | 1.130 (0.971, 1.316) | 1.340 (0.940, 1.910) |

*Notes.* HR, hazard ratio; CI, confidence interval; PM_2.5_, particulate matter with a diameter of 2.5 μm (micrometres) or smaller; NO_2_, nitrogen dioxide; ppb, parts per billion. Estimates adjusted for confounders. Inverse probability weights were used to account for attrition bias. Statistically significant effects are in bold. 4154 observations. 264 and 345 transitions to dementia in Scenario 1 and 2 models, respectively. Environmental correlates of transitions from no dementia to death and from dementia to death are not reported because they were not the focus of the study.

**Table S8.** Neighbourhood environmental attributes as correlates of transitions from normal cognition to mild cognitive impairment (MCI): overall- and independent-effect models for Scenario 1 and 2 (N = 1036 followed up for ~6 years)

|  |  | Scenario 1 (optimistic) | | Scenario 2 (pessimistic) | |
| --- | --- | --- | --- | --- | --- |
| Neighbourhood environmental attribute |  | Overall-effect models | Independent-effect models | Overall-effect models | Independent-effect models |
|  | Spatial indicator | HR (95% CI) | HR (95% CI) | HR (95% CI) | HR (95% CI) |
| Population density (100 persons/km^2^) | Original | 0.996 (0.987, 1.004) | 1.001 (0.991, 1.012) | 0.997 (0.988, 1.006) | 0.998 (0.986, 1.009) |
|  | Cumulative | 0.993 (0.983, 1.002) | 0.997 (0.985, 1.010) | 0.997 (0.987, 1.007) | 0.998 (0.984, 1.012) |
| Street intersection density (intersections/km^2^) | Original | **0.992 (0.985, 0.998)** | **0.990 (0.982, 0.998)** | 0.999 (0.993, 1.005) | 0.995 (0.988, 1.001) |
|  | Cumulative | 0.996 (0.990, 1.003) | **0.993 (0.986, 0.999)** | 1.000 (0.993, 1.006) | 0.997 (0.991, 1.003) |
| Transit points (count) | Original | 0.999 (0.989, 1.009) | 1.009 (0.996, 1.023) | 1.001 (0.991, 1.010) | 1.007 (0.993, 1.020) |
|  | Cumulative | 1.000 (0.990, 1.010) | 1.013 (0.999, 1.028) | 1.002 (0.992, 1.012) | 1.009 (0.995, 1.024) |
| Commercial land (%) | Original | **0.978 (0.955, 0.999)** | **0.961 (0.927, 0.997)** | **0.983 (0.966, 0.999)** | **0.960 (0.927, 0.996)** |
|  | Cumulative | 0.979 (0.957, 1.002) | **0.964 (0.929, 0.999)** | **0.980 (0.961, 0.998)** | **0.957 (0.924, 0.992)** |
| Parkland (%) | Original | 1.012 (0.995, 1.029) | 1.013 (0.993, 1.034) | 1.008 (0.990, 1.027) | 1.007 (0.986, 1.027) |
|  | Cumulative | **1.020 (1.000, 1.039)** | 1.017 (0.995, 1.039) | 1.012 (0.990, 1.035) | 1.011 (0.989, 1.033) |
| Tree cover (%) | Original | 0.987 (0.962, 1.012) | 0.976 (0.944, 1.008) | 0.983 (0.954, 1.013) | 0.979 (0.943, 1.015) |
|  | Cumulative | 0.989 (0.963, 1.015) | 0.989 (0.942, 1.038) | 0.981 (0.948, 1.014) | 0.982 (0.939, 1.027) |
| Blue space (%) | Original | 1.006 (0.996, 1.015) | 1.007 (0.994, 1.020) | 1.001 (0.991, 1.010) | 1.004 (0.991, 1.017) |
|  | Cumulative | 1.006 (0.996, 1.016) | 1.014 (0.999, 1.027) | 1.000 (0.991, 1.010) | 1.005 (0.991, 1.020) |
| NO_2_ (ppb) | Original | 0.968 (0.858, 1.091) | 1.072 (0.912, 1.259) | 1.047 (0.941, 1.164) | 1.137 (0.971, 1.331) |
|  | Cumulative | 0.985 (0.862, 1.125) | 1.079 (0.912, 1.275) | 1.044 (0.928, 1.174) | 1.138 (0.967, 1.339) |
| PM_2.5_ (µg/m^3^) | Original | 0.979 (0.583, 1.659) | 1.027 (0.898, 1.190) | 0.824 (0.499, 1.361) | 0.666 (0.369, 1.197) |
|  | Cumulative | 0.992 (0.590, 1.694) | 1.083 (0.955, 1.250) | 0.888 (0.506, 1.560) | 0.729 (0.364, 1.459) |

*Notes.* HR, hazard ratio; CI, confidence interval; PM_2.5_, particulate matter with a diameter of 2.5 μm (micrometres) or smaller; NO_2_, nitrogen dioxide; ppb, parts per billion. Estimates adjusted for confounders. Inverse probability weights were used to account for attrition bias. Statistically significant effects are in bold. 2530 observations. 300 and 316 transitions from normal cognition to MCI in Scenario 1 and 2 models, respectively.

**Table S9.** Neighbourhood environmental attributes as correlates of transitions from normal cognition to dementia: overall- and independent-effect models for Scenario 1 and 2 (N = 1036 followed up for ~6 years)

|  |  | Scenario 1 (optimistic) | | Scenario 2 (pessimistic) | |
| --- | --- | --- | --- | --- | --- |
| Neighbourhood environmental attribute |  | Overall-effect models | Independent-effect models | Overall-effect models | Independent-effect models |
|  | Spatial indicator | HR (95% CI) | HR (95% CI) | HR (95% CI) | HR (95% CI) |
| Population density (100 persons/km^2^) | Original | 1.023 (0.981, 1.068) | **0.997 (0.995, 0.999)** | 1.012 (0.953, 1.074) | **0.871 (0.795, 0.953)** |
|  | Cumulative | 1.030 (0.973, 1.090) | **0.987 (0.979, 0.996)** | 1.001 (0.929, 1.079) | **0.962 (0.927, 0.998)** |
| Street intersection density (intersections/km^2^) | Original | **1.041 (1.015, 1.068)** | 1.055 (0.944, 1.179) | **1.062 (1.030, 1.096)** | 0.985 (0.947, 1.025) |
|  | Cumulative | **1.027 (1.002, 1.053)** | **0.993 (0.988, 0.998)** | **1.041 (1.008, 1.075)** | **0.956 (0.917, 0.997)** |
| Transit points (count) | Original | 1.055 (0.999, 1.114) | 1.082 (0.959, 1.221) | **1.104 (1.016, 1.200)** | 1.054 (0.956, 1.162) |
|  | Cumulative | 1.045 (0.992, 1.102) | 1.032 (0.962, 1.106) | 1.054 (0.960, 1.158) | 1.222 (0.816, 1.829) |
| Commercial land (%) | Original | 1.037 (0.985, 1.091) | **0.979 (0.960, 0.999)** | 1.043 (0.966, 1.126) | 0.949 (0.880, 1.023) |
|  | Cumulative | **1.071 (1.000, 1.146)** | **0.994 (0.989, 0.998)** | **1.153 (1.044, 1.272)** | 0.997 (0.972, 1.023) |
| Parkland (%) | Original | 0.998 (0.886, 1.124) | 0.865 (0.693, 1.080) | 0.947 (0.764, 1.174) | 0.903 (0.753, 1.082) |
|  | Cumulative | 1.004 (0.837, 1.203) | 0.931 (0.831, 1.042) | 0.964 (0.325, 2.853) | 1.040 (0.808, 1.338) |
| Tree cover (%) | Original | 0.992 (0.963, 1.023) | 1.032 (0.803, 1.326) | 0.856 (0.686, 1.068) | 1.014 (0.782, 1.314) |
|  | Cumulative | 0.987 (0.953, 1.021) | 1.168 (0.878, 1.553) | 0.892 (0.650, 1.225) | 1.266 (0.651, 2.465) |
| Blue space (%) | Original | 0.896 (0.724, 1.109) | 1.069 (0.957, 1.194) | 0.860 (0.676, 1.095) | 1.048 (0.905, 1.213) |
|  | Cumulative | 0.908 (0.767, 1.074) | 0.970 (0.835, 1.127) | 0.679 (0.428, 1.078) | 1.151 (0.839, 1.578) |
| NO_2_ (ppb) | Original | 1.521 (0.630, 3.669) | 1.758 (0.543, 5.681) | **4.010 (1.244, 12.928)** | **3.578 (1.420, 9.016)** |
|  | Cumulative | **3.212 (1.066, 9.678)** | **4.452 (1.374, 14.427)** | **6.670 (1.429, 31.125)** | **5.212 (1.373, 19.785)** |
| PM_2.5_ (µg/m^3^) | Original | 1.346 (0.709, 2.555) | 1.403 (0.658, 2.992) | 2.533 (0.602, 10.658) | 1.061 (0.571, 1.971) |
|  | Cumulative | 1.430 (0.748, 2.734) | 2.051 (0.733, 5.739) | 3.083 (0.664, 14.315) | 3.346 (0.602, 18.000) |

*Notes.* HR, hazard ratio; CI, confidence interval; PM_2.5_, particulate matter with a diameter of 2.5 μm (micrometres) or smaller; NO_2_, nitrogen dioxide; ppb, parts per billion. Estimates adjusted for confounders. Inverse probability weights were used to account for attrition bias. Statistically significant effects are in bold. 2530 observations. 44 and 14 transitions from normal cognition to dementia in Scenario 1 and 2 models, respectively.

**Table S10.** Neighbourhood environmental attributes as correlates of transitions from mild cognitive impairment (MCI) to dementia: overall- and independent-effect models for Scenario 1 and 2 (N = 1036 followed up for ~6 years)

|  |  | Scenario 1 (optimistic) | | Scenario 2 (pessimistic) | |
| --- | --- | --- | --- | --- | --- |
| Neighbourhood environmental attribute |  | Overall-effect models | Independent-effect models | Overall-effect models | Independent-effect models |
|  | Spatial indicator | HR (95% CI) | HR (95% CI) | HR (95% CI) | HR (95% CI) |
| Population density (100 persons/km^2^) | Original | 0.998 (0.982, 1.014) | 1.022 (0.997, 1.045) | 0.998 (0.983, 1.013) | 1.016 (0.997, 1.035) |
|  | Cumulative | 0.990 (0.968, 1.013) | 1.015 (0.993, 1.037) | 0.994 (0.976, 1.011) | 1.004 (0.972, 1.037) |
| Street intersection density (intersections/km^2^) | Original | **0.990 (0.982, 0.998)** | 0.996 (0.984, 1.008) | **0.994 (0.989, 0.999)** | 0.998 (0.987, 1.009) |
|  | Cumulative | **0.991 (0.982, 0.999)** | 0.997 (0.981, 1.013) | **0.993 (0.988, 0.998)** | 0.998 (0.985, 1.011) |
| Transit points (count) | Original | **0.966 (0.948, 0.984)** | **0.964 (0.940, 0.990)** | **0.974 (0.957, 0.991)** | **0.972 (0.950, 0.996)** |
|  | Cumulative | **0.969 (0.951, 0.987)** | **0.971 (0.946, 0.997)** | **0.977 (0.959, 0.994)** | **0.979 (0.959, 0.999)** |
| Commercial land (%) | Original | **0.965 (0.931, 0.999)** | 0.966 (0.904, 1.032) | 0.975 (0.944, 1.008) | 0.967 (0.905, 1.033) |
|  | Cumulative | **0.960 (0.923, 0.998)** | 0.964 (0.906, 1.026) | 0.974 (0.943, 1.007) | 0.968 (0.909, 1.031) |
| Parkland (%) | Original | 1.017 (0.990, 1.044) | 1.013 (0.982, 1.044) | 1.021 (0.995, 1.048) | 1.013 (0.982, 1.045) |
|  | Cumulative | 1.015 (0.988, 1.043) | 1.000 (0.967, 1.087) | 1.007 (0.984, 1.030) | 1.017 (0.990, 1.045) |
| Tree cover (%) | Original | 0.975 (0.943, 1.008) | 0.976 (0.913, 1.043) | **0.962 (0.932, 0.993)** | 0.975 (0.914, 1.040) |
|  | Cumulative | **0.970 (0.943, 0.998)** | 0.986 (0.916, 1.061) | **0.955 (0.917, 0.995)** | 0.979 (0.911, 1.052) |
| Blue space (%) | Original | 1.003 (0.988, 1.018) | 0.986 (0.966, 1.007) | 1.002 (0.987, 1.017) | 0.992 (0.973, 1.012) |
|  | Cumulative | 1.004 (0.990, 1.019) | 0.994 (0.974, 1.014) | 1.004 (0.990, 1.019) | 0.998 (0.975, 1.022) |
| NO_2_ (ppb) | Original | 1.017 (0.828, 1.249) | 1.017 (0.828, 1.249) | 1.124 (0.901, 1.402) | 0.991 (0.737, 1.332) |
|  | Cumulative | 0.962 (0.777, 1.191) | 0.962 (0.777, 1.191) | 1.141 (0.887, 1.468) | 1.223 (0.861, 1.481) |
| PM_2.5_ (µg/m^3^) | Original | 2.030 (0.391, 10.549) | 2.567 (0.913, 7.216) | 1.159 (0.541, 2.481) | 2.191 (0.685, 7.007) |
|  | Cumulative | 1.180 (0.491, 2.838) | 1.180 (0.491, 2.838) | 1.018 (0.466, 2.223) | 0.969 (0.325, 2.893) |

*Notes.* HR, hazard ratio; CI, confidence interval; PM_2.5_, particulate matter with a diameter of 2.5 μm (micrometres) or smaller; NO_2_, nitrogen dioxide; ppb, parts per billion. Estimates adjusted for confounders. Inverse probability weights were used to account for attrition bias. Statistically significant effects are in bold. 2530 observations. 60 and 90 transitions from MCI to dementia in Scenario 1 and 2 models, respectively.

**Table S11.** Neighbourhood environmental attributes as correlates of reversal from mild cognitive impairment (MCI) to normal cognition: overall- and independent-effect models for Scenario 1 and 2 (N = 1036 followed up for ~6 years)

|  |  | Scenario 1 (optimistic) | | Scenario 2 (pessimistic) | |
| --- | --- | --- | --- | --- | --- |
| Neighbourhood environmental attribute |  | Overall-effect models | Independent-effect models | Overall-effect models | Independent-effect models |
|  | Spatial indicator | HR (95% CI) | HR (95% CI) | HR (95% CI) | HR (95% CI) |
| Population density (100 persons/km^2^) | Original | 1.001 (0.990, 1.011) | 1.000 (0.987, 1.014) | 0.997 (0.988, 1.006) | 0.996 (0.984, 1.007) |
|  | Cumulative | 1.001 (0.989, 1.012) | 1.001 (0.986, 1.016) | 0.996 (0.987, 1.007) | 0.996 (0.984, 1.009) |
| Street intersection density (intersections/km^2^) | Original | 0.997 (0.991, 1.004) | 0.998 (0.985, 1.011) | 0.998 (0.991, 1.004) | 0.999 (0.995, 1.003) |
|  | Cumulative | 0.997 (0.990, 1.004) | 0.997 (0.987, 1.006) | 0.999 (0.993, 1.005) | 1.000 (0.990, 1.010) |
| Transit points (count) | Original | 1.003 (0.991, 1.015) | 1.015 (0.999, 1.031) | 1.004 (0.992, 1.015) | **1.018 (1.003, 1.032)** |
|  | Cumulative | 1.003 (0.991, 1.015) | **1.018 (1.002, 1.035)** | 1.005 (0.994, 1.017) | **1.022 (1.007, 1.038)** |
| Commercial land (%) | Original | 0.981 (0.956, 1.006) | 0.974 (0.935, 1.014) | 0.979 (0.955, 1.004) | 0.963 (0.927, 1.001) |
|  | Cumulative | 0.979 (0.953, 1.005) | 0.979 (0.940, 1.019) | 0.979 (0.955, 1.004) | **0.961 (0.924, 0.999)** |
| Parkland (%) | Original | 1.009 (0.990, 1.029) | 1.007 (0.985, 1.030) | 1.009 (0.991, 1.027) | 1.003 (0.983, 1.023) |
|  | Cumulative | 1.012 (0.991, 1.033) | 1.012 (0.988, 1.036) | 1.011 (0.993, 1.030) | 1.007 (0.986, 1.028) |
| Tree cover (%) | Original | 0.993 (0.954, 1.033) | 1.000 (0.950, 1.054) | 0.987 (0.952, 1.022) | 0.987 (0.947, 1.029) |
|  | Cumulative | 1.000 (0.958, 1.044) | 1.037 (0.980, 1.098) | 0.980 (0.942, 1.020) | 0.998 (0.949, 1.049) |
| Blue space (%) | Original | 1.006 (0.995, 1.017) | **1.011 (1.000, 1.021)** | 1.006 (0.996, 1.017) | **1.015 (1.002, 1.028)** |
|  | Cumulative | 1.007 (0.996, 1.019) | **1.017 (1.002, 1.033)** | 1.007 (0.996, 1.017) | **1.018 (1.004, 1.032)** |
| NO_2_ (ppb) | Original | 0.943 (0.806, 1.104) | 1.024 (0.834, 1.257) | 0.993 (0.865, 1.142) | 1.111 (0.929, 1.330) |
|  | Cumulative | 0.938 (0.789. 1.116) | 0.991 (0.803. 1.225) | 0.910 (0.728, 1.138) | 1.105 (0.919, 1.329) |
| PM_2.5_ (µg/m^3^) | Original | 0.933 (0.529, 1.648) | 0.832 (0.411, 1.687) | 0.758 (0.460, 1.250) | 0.727 (0.390, 1.354) |
|  | Cumulative | 1.248 (0.680, 2.291) | 1.339 (0.611, 2.934) | 0.892 (0.488, 1.631) | 1.035 (0.481, 2.223) |

*Notes.* HR, hazard ratio; CI, confidence interval; PM_2.5_, particulate matter with a diameter of 2.5 μm (micrometres) or smaller; NO_2_, nitrogen dioxide; ppb, parts per billion. Estimates adjusted for confounders. Inverse probability weights were used to account for attrition bias. Statistically significant effects are in bold. 2530 observations. 222 and 263 transitions from MCI to normal cognition in Scenario 1 and 2 models, respectively.

***References***

[1] Adams MA, Frank LD, Schipperijn J, Smith G, Chapman J, Christiansen LB, et al. International variation in neighborhood walkability, transit, and recreation environments using geographic information systems: the IPEN adult study. Int J Health Geogr. 2014;13:43.

[2] Frank LD, Fox EH, Ulmer JM, Chapman JE, Kershaw SE, Sallis JF, et al. International comparison of observation-specific spatial buffers: maximizing the ability to estimate physical activity. Int J Health Geogr. 2017;16(1):4.

[3] ArcGIS Desktop. Redlands: C.E.S.R.I.; Release 10.6. 2021.

[4] Cerin E, Chan YK, Symmons M, Soloveva M, Martino E, Shaw JE, et al. Associations of the neighbourhood built and natural environment with cardiometabolic health indicators: A cross-sectional analysis of environmental moderators and behavioural mediators. Environ Res. 2024;240(Pt 2):117524.

[5] Gunn LD, King TL, Mavoa S, Lamb KE, Giles-Corti B, Kavanagh A. Identifying destination distances that support walking trips in local neighborhoods. J Transp Health. 2017;5:133-41.

[6] Cerin E, Van Dyck D, Zhang CJP, Van Cauwenberg J, Lai PC, Barnett A. Urban environments and objectively-assessed physical activity and sedentary time in older Belgian and Chinese community dwellers: potential pathways of influence and the moderating role of physical function. Int J Behav Nutr Phys Act. 2020;17(1):73.

[7] Cochrane T, Yu Y, Davey R, Cerin E, Cain KL, Conway TL, et al. Associations of built environment and proximity of food outlets with weight status: Analysis from 14 cities in 10 countries. Prev Med. 2019;129:105874.

[8] Australian Bureau of Statistics. Socio-Economic Indexes for Areas (SEIFA) Australia 2021 [cited 2024 December 24]. Available from: <https://www.abs.gov.au/statistics/people/people-and-communities/socio-economic-indexes-areas-seifa-australia/latest-release>. .

[9] Knibbs LD, van Donkelaar A, Martin RV, Bechle MJ, Brauer M, Cohen DD, et al. Satellite-Based Land-Use Regression for Continental-Scale Long-Term Ambient PM(2.5) Exposure Assessment in Australia. Environ Sci Technol. 2018;52(21):12445-55.

[10] Knibbs LD, Hewson MG, Bechle MJ, Marshall JD, Barnett AG. A national satellite-based land-use regression model for air pollution exposure assessment in Australia. Environ Res. 2014;135:204-11.

[11] Knibbs LD, Coorey CP, Bechle MJ, Marshall JD, Hewson MG, Jalaludin B, et al. Long-term nitrogen dioxide exposure assessment using back-extrapolation of satellite-based land-use regression models for Australia. Environ Res. 2018;163:16-25.

[12] Ahmed SM, Mishra GD, Moss KM, Yang IA, Lycett K, Knibbs LD. Maternal and Childhood Ambient Air Pollution Exposure and Mental Health Symptoms and Psychomotor Development in Children: An Australian Population-Based Longitudinal Study. Environ Int. 2022;158:107003.

[13] Zhang J, McLaughlin SJ, Li LW. Cumulative exposure to air pollution and subsequent mortality among older adults in China. J Public Health (Oxf). 2019;41(3):518-26.

[14] Hindmarch I, Lehfeld H, de Jongh P, Erzigkeit H. The Bayer Activities of Daily Living Scale (B-ADL). Dement Geriatr Cogn Disord. 1998;9 Suppl 2:20-6.

[15] Lawton MP, Brody EM. Assessment of older people: self-maintaining and instrumental activities of daily living. Gerontologist. 1969;9(3):179-86.

[16] Yesavage JA, Brink TL, Rose TL, Lum O, Huang V, Adey M, et al. Development and validation of a geriatric depression screening scale: a preliminary report. J Psychiatr Res. 1982;17(1):37-49.

[17] Wood SN. Generalised additive models: An introduction with R. (2nd ed). Boca Raton, FL: Chapman & Hall/CRC; 2017.

[18] Burnham KP, Anderson DR. Model selection and multimodel inference: a practical information-theoretic approach. 2nd ed. New York: Springer Verlag; 2002.
